# Interleukin-18 binding protein deficiency results in gut microbiota dysbiosis and aggravated diet-induced MASH in mice

**DOI:** 10.1016/j.jhepr.2025.101629

**Published:** 2025-10-10

**Authors:** Emmanuel Somm, Elodie Perroud, Yunju Jo, Karina Lindner, Frédérique Ino, Sophie A. Montandon, Christelle Veyrat-Durebex, Franck Bontems, Florian Visentin, Nadia Gaïa, Vladimir Lazarevic, Anne-Claude Gavin, Jacques Schrenzel, Dongryeol Ryu, Karim Gariani, Cem Gabay, François R. Jornayvaz

**Affiliations:** 1 Service of Endocrinology, Diabetes and Metabolism, Department of Medicine, Geneva University Hospitals/University of Geneva, Geneva, Switzerland; 2 Diabetes Center, Faculty of Medicine, University of Geneva, Geneva, Switzerland; 3 Department of Cell Physiology and Metabolism, Faculty of Medicine, University of Geneva, Geneva, Switzerland; 4 Department of Biomedical Science and Engineering, Gwangju Institute of Science and Technology, Gwangju, South Korea; 5 Molecular and Integrative Biology (MIB) Lab, Sungkyunkwan University School of Medicine, Suwon, South Korea; 6 Genomic Research Laboratory, Department of Medicine, Geneva University Hospitals/University of Geneva, Geneva, Switzerland; 7 Bacteriology Laboratory, Department of Diagnostics, Geneva University Hospitals, Geneva, Switzerland; 8 Department of Pathology and Immunology, Faculty of Medicine, University of Geneva, Geneva, Switzerland

**Keywords:** MASLD, MASH, Interleukin-18 binding protein, Gut microbiota, Fibrosis

## Abstract

**Background & Aims:**

Metabolic dysfunction-associated steatotic liver disease (MASLD)/metabolic dysfunction-associated steatohepatitis (MASH) are now the most prevalent hepatic disorders worldwide. Growing evidence implicates physiological alterations in the gut–liver axis and gut microbiota dysbiosis in this process. IL-18-binding protein (IL-18BP) forms high affinity complexes with IL-18, thus blocking its interaction with IL-18 receptors.

**Methods:**

We used high-fat diet (HFD) and methionine choline deficient (MCD) diet to model MASLD/MASH in wild-type (WT) male mice (n = 6–8 mice per group). We also studied antimicrobial peptides (AMPs) production, gut microbiota composition, and liver phenotype in *Il18bp*^*-/-*^ male mice on both HFD and MCD diets (n = 5–7 mice per group). We manipulated gut microbiota of *Il18bp*^*-/-*^ and WT male mice through administration of phages, antibiotics, and through co-housing experiments (n = 4–8 mice per group).

**Results:**

Feeding WT mice with a HFD or an MCD diet led to a decrease in ileal AMPs expressions (respectively, by 63% and 37% for *Lyz1*; by 47% and 84% for *Ang4*; by 86% and 46% for *Pla2g2a*) and an enrichment in gut proteobacteria (respectively, by 7- and 23-fold for α-proteobacteria; by 2.1- and 1.7-fold for δ-proteobacteria; by 14- and 20-fold for γ-proteobacteria) when compared with standard chow diet (*p* <0.05). These changes were associated with a reduction in the ileal *Il18bp* expression (respectively, by 77% and 46%) in HFD and MCD diet-fed WT mice *vs.* chow diet-fed WT mice (*p* <0.05). *Il18bp*^*-/-*^ mice exhibited a decrease in gut AMPs expression and storage (AMP granules area/crypt respectively decreased by 57% and 62.5% in *Il18bp*^*-/-*^*vs.* WT mice on HFD and MCD diets). Moreover, *Clostridium/Turicimonas/Escherichia* bacteria were constitutively over-represented in gut microbiota of *Il18bp*^*-/-*^*vs.* WT mice in a diet-amplified manner. Compared with WT mice, *Il18bp*^*-/-*^ mice exhibited increased diet-induced hepatic damage (circulating alanine aminotransferase 84 *vs.* 41 U/L on HFD; 1,218 *vs.* 738 U/L on MCD diet, *p* <0.05), inflammation (liver tumor necrosis factor-alpha content 72 *vs.* 35 pg/g protein on HFD; 441 *vs.* 169 pg/g protein on MCD diet, *p* <0.05), and fibrosis (Sirius red 1.45 *vs.* 0.36% on HFD; 1.64 *vs.* 0.65% on MCD diet, *p* <0.01). These changes occurred independently of steatosis modification. Phages, antibiotic, and co-housing experiments revealed that specific gut microbiota featuring *Il18bp*^*-/-*^ mice is implicated in their exacerbated liver inflammation and fibrosis status.

**Conclusions:**

IL-18BP limits the progression of MASLD/MASH by maintaining normal intestinal production of AMPs and composition of the gut microbiota.

**Impact and implications:**

We presently highlight a previously unknown protective role of IL-18BP in the integrity of the gut-liver axis. Increasing IL-18-binding protein levels (a clinically validated option to treat rare systemic auto-inflammatory diseases) represents a novel therapeutic perspective, not only for patients with MASLD/MASH, but also for patients presenting gut microbiota dysbiosis.

## Highlights

• Intestinal antimicrobial peptides and IL-18BP are decreased while gut proteobacteria are increased in WT mice on HFD and MCD diet.
• IL-18BP deficiency alters antimicrobial peptides production on HFD and MCD diet.
• IL-18BP deficiency enriches gut microbiota in *Escherichia/Turicimonas/Clostridium* in a diet-amplified manner.
• IL-18BP deficiency worsens hepatic inflammation and fibrosis on HFD and MCD diet.
• Gut microbiota is implicated in the exacerbated liver inflammation and fibrosis observed in IL-18BP-deficient mice.

## Graphical abstract

**Figure:**
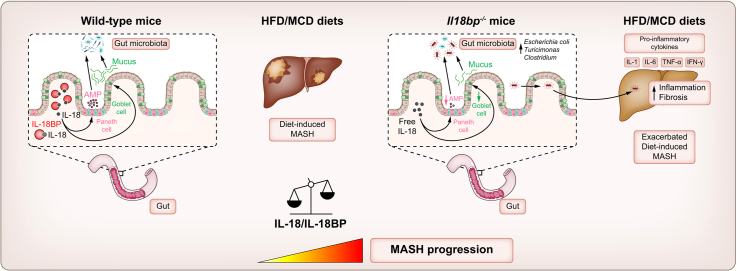

## Introduction

Metabolic dysfunction-associated steatotic liver disease (MASLD) is a growing public health concern.^1^ A significant proportion of patients with MASLD experience a state of hepatic inflammation now named metabolic dysfunction-associated steatohepatitis (MASH), which can result in hepatic fibrosis, cirrhosis, and even hepatocellular carcinoma (HCC).^1^ Although liver fat storage is quite reversible, fibrosis often represents a critical step in disease progression.^1^ Although lifestyle changes have beneficial effects, efficient therapies to treat MASLD/MASH are lacking.^1^

The gut–liver axis (i.e. transport of gut-derived products directly to the liver, allowing nutrients absorption while limiting the dissemination of microbes and toxins to the systemic circulation) has recently emerged as a priority field of investigation in the context of MASLD/MASH.^2^ Dietary nutrients or pollutants, integrity of the gut barrier, gut microbiota composition and its byproducts, as well as local immunity and antimicrobial peptides (AMPs) production determine an integrated host–microbe interaction, which preserves physiological homeostasis or triggers pathologies in case of dysfunction.

IL-18 is a member of the IL-1 superfamily of cytokines.^3^ IL-18 precursor is processed by inflammasome/caspase-1 into a mature and biologically active form that binds to its specific receptor composed of two chains (IL-18Rα and IL-18Rβ).^3^^,^^4^ This interaction triggers an intracellular signaling pathway ultimately leading to activation of nuclear factor kappa B, interferon-gamma (IFN-γ) and inflammatory processes driving the Th1 immune response,^3^^,^^4^ The soluble IL-18-binding protein (IL-18BP) binds circulating IL-18 with high affinity, leaving only a small fraction of free IL-18 able to trigger receptor-mediated signaling.^3^^,^^4^ IL-18BP is thus considered as a negative regulator of IL-18 signaling.

The role of IL-18 signaling in inflammatory and infectious diseases is well established.^3^^,^^4^ However, recent findings, in particular experimental studies involving genetically modified mice, also implicate IL-18 signaling in metabolism (as recently reviewed^5^). Initially, IL-18 deficient (*Il18*^*-/-*^) mice have been described as hyperphagic and obese, exhibiting secondary hepatic insulin resistance.^6^ Another study showed that *Il18*^*-/-*^ mice develop hypercholesterolemia and hypertriglyceridemia before the manifestation of obesity.^7^ Finally, other works have shown that *Il18*^*-/-*^ mice exhibit an impaired intestinal barrier integrity,^8^^,^^9^ and gut dysbiosis involved in MASLD progression.^10^

The role of IL-18BP in the gut–liver axis and MASLD/MASH contexts has not been yet identified and is the main goal of our study.

## Materials and methods

### Animals

All experimental protocols were performed in accordance with the Swiss animal welfare laws. Wild-type (WT) (C57BL/6J) and *Il18bp*^*-/-*^ mice (generated as previously described^11^), originating from our own breeding colonies, were housed in standard conditions in the animal facility of the Geneva Medical Center. All WT and *Il18bp*^*-/-*^ male mice used in experiments originated from WT and *Il18bp*^*-/-*^ genitors housed separately to avoid gut microbiota exchange between genotypes. Il18bp-tomato^ki/ki^ (knock-in) reporter mice (harboring fluorescence in nuclei of IL-18BP-producing cells) were generated as previously reported.^12^

### Diets

Starting at 10 weeks of age, WT and *Il18bp*^*-/-*^ male mice were fed a chow diet (Safe 150, Safe, Augy, France) for 10 weeks more, or, to model MASLD/MASH, were fed either (i) high-fat diet (HFD) (Research Diets#D12492, Research Diets, New Brunswick, NJ, USA) (60 kcal%fat) for 10 weeks or (ii) methionine choline-deficient (MCD)-diet (Research Diets#A02082002BR, Research Diets, New Brunswick, NJ, USA) for 7 weeks.

### Treatment

For phages treatment, mice were hand fed with 100 μl of *E. coli*-*Proteus* bacteriophage solution (Microgen, Moscow, Russia) two times per week during 7 weeks of MCD diet consumption or 6 weeks of HFD consumption (Fig. S8). For antibiotic treatment, mice received ampicillin (Ratiopharm, Ulm, Germany) via drinking water at a concentration of 1 g/L, starting 1 week before and during all 7 weeks of MCD diet consumption (Fig. S7) or 6 weeks of HFD consumption (Fig. S9) (water/antibiotic renewal every 3 days). For the co-housing experiment, WT and *Il18bp*^*-/-*^ mice were grouped immediately at weaning and during all 7 weeks of MCD diet consumption or 6 weeks of HFD consumption (Fig. S10).

Only male mice of similar age (17–24 weeks) were analyzed, allowing to avoid confounding impact of aging and hormonal variations. At the end of all experiments, all mice were fasted for 3 h, slightly anesthetized with isoflurane and immediately sacrificed. Blood samples were collected in EDTA-coated tubes and stored at -80 °C, and organs were dissected and weighed before fixation and cryopreservation in liquid nitrogen.

### Metabolic phenotyping

Body composition was determined using magnetic resonance imaging (EchoMedicalSystems, Houston, TX, USA). Indirect calorimetry was performed using the standard procedures of the LabMaster system (TSE Systems, Berlin, Germany). For the intraperitoneal glucose tolerance test (GTT), 2 mg of glucose/g body weight was given after a 6-h fast and glycemia was measured at each indicated time-point with an Accu-Chek glucose meter and strips (Roche Diagnostics, Rotkreuz, Zug, Switzerland). For the intraperitoneal insulin tolerance test (ITT), 0.75 mU of insulin/g body weight (NovoRapid, NovoNordisk, Bagsvaerd, Denmark) was given after a 3-h fast and glycemia was measured at each indicated time-point with an Accu-Chek glucose meter and strips (Roche Diagnostics, Rotkreuz, Zug, Switzerland).

### Blood and tissue metabolites and cytokines analyses

Plasma levels of alanine aminotransferase (ALT), aspartate aminotransferase, triglycerides, and cholesterol were assessed using a Cobas C111 robot and supplied reagents (Roche Diagnostics, Rotkreuz, Zug, Switzerland). For hepatic triglyceride and cholesterol content, total lipids were extracted using methyl tert-butyl ether before quantification using a Cobas C111 robot. Blood and liver cytokines content were measured using the mouse V-Plex Proinflammatory Panel kit and the QuickPlex MSD SQ120 instrument from MesoScale Discovery (MSD, Rockville, MD, USA), following the manufacturer's instructions. Blood lipopolysaccharide (LPS) content was measured using the Mouse Lipopolysaccharides ELISA Kit CSB-E13066m (Cusabio, Houston, TX, USA).

### Gene expression

Total RNA isolated from liver or gut (ileum) samples using TRI Reagent Solution (ThermoFisher, Waltham, MA, USA) were reverse-transcribed using the MMLV kit (ThermoFisher, Waltham, MA, USA). cDNAs were quantified by real-time PCR using Power SYBR Green mix and the Light-Cycler 480 Detection System (Roche Diagnostics, Rotkreuz, Zug, Switzerland), normalized using the housekeeping gene Rps29, folded to the mean value of the corresponding control group and expressed as arbitrary units (A.U). For bulk RNA sequencing analysis, RNA integrity was controlled with a 2100 Bioanalyzer (Agilent, Santa Clara, CA, USA), before sequencing with an Illumina NovaSeq 6000 (Illumina, San Diego, CA, USA) at the iGE3 Genomics Platform of Geneva University. The RNA-seq data were processed and analyzed using R (version 4.3.2) and R studio (version 2023.03.0 Build 386; RStudio, Inc., Boston, MA, USA).

### Histology/immunohistochemistry

For histology, liver and intestine were either fixed overnight in 10% formalin before dehydration and embedded in paraffin or immediately embedded in OCT medium (Cell path LTD, Newtown, Powys, UK) and frozen on solid CO_2_ before storage at -80 °C. H&E, Sirius red (SR), Lendrum’s phloxine–tartrazine, periodic acid–Schiff (PAS) and Oil red O (ORO) staining were performed using classical procedures. Pictures were acquired using an EVOS FL digital inverted fluorescence microscope (ThermoFisher, Waltham, MA, USA), Axiophot microscope and an Axiocam color camera (Zeiss, Oberkochen, Germany) or an Axio Scan.Z1 slide scanner (Zeiss, Oberkochen, Germany). For immunohistochemistry, paraffin sections were dewaxed, and rehydrated using xylene/ethanol baths and then heated at 95 °C in a 10 mM/pH 6.0 sodium citrate bath for 10 min. The liver sections were incubated overnight at 4 °C with primary antibodies diluted in PBS/0.1% BSA, washed in PBS, and incubated for 1 h with a secondary antibody (Alexa Fluor; Fluor Corporation, Irving, TX, USA) diluted (1:1,000) in PBS/0.1% BSA.

For histomorphometry, SR staining (fibrosis) was evaluated in four representative images per animal using Image J software (Image J, Washington, DC, USA). Large vessels, exhibiting a massive confounding collagen staining, were excluded from the analysis which represents only collagen fibers emerging in the liver parenchyma. Areas of antimicrobial peptide granules in ileal crypts were quantified using the open access QuPath software (Open Software for Bioimage Analysis; https://qupath.github.io). AMP granules/crypts area were evaluated in 25–35 independents region originating from 4 to 5 WT or *Il18bp*^*-/-*^ mice per group. Goblet cells/villus were manually evaluated in 20–30 villus/animal from four WT or *Il18bp*^*-/-*^ mice per group. The number of inflammatory foci (per 200 × field) were assessed/counted manually (in four independent fields per animal), as clinically determined to evaluate the inflammatory component of the MASLD activity (NAS) score.^13^ ORO staining (neutral lipids content) was quantified in one representative image per animal using Image J software (Image J, Washington, DC, USA).

### Microbiota analysis

For gut microbiota analysis by next generation sequencing, DNA from colonic feces was extracted using Quick-DNA Fecal/Soil Microbe Miniprep Kit (Zymo Research, Irvine, CA, USA) with 20 min bead beating on a Vortex Genie 2 (Scientific Industries, Bohemia, NY, USA) with a horizontal microtube adapter and stored at -20 °C. The V3–4 region of the bacterial 16S rRNA gene was amplified using 1 ng of DNA using ZymoBIOMICS PCR PreMix (Zymo Research, Irvine, CA, USA). Duplicate PCRs of each sample were combined and run on a 2100 Bioanalyzer (Agilent Technologies, Santa Clara, CA, USA) for quality analysis and quantification. The Illumina MiSeq 2 × 300 sequencing was performed using the MetaFast protocol (Fasteris, Geneva, Switzerland). Sequencing data were submitted to the European Nucleotide Archive (ENA; www.ebi.ac.uk/ena; study number: PRJEB60286). Forward and reverse reads were paired-end-joined with PEAR v.0.9.11. The merged sequences were subjected to the USEARCH v.11.0.667 package UNOISE3 pipeline for clustering into zero-radius operational taxonomic units (zOTUs). Taxonomic assignment of the representative zOTUs was performed using MOTHUR version 1.43.0 and the EzBioCloud 16S rRNA gene sequence database. zOTUs were checked to have at least 90% identity to reference prokaryotic EzBioCloud 16S sequences using the USEARCH algorithm from the USEARCH package. Four zOTUs with the average relative abundance >0.5% in the two negative extraction controls were removed. Before calculating the Shannon species diversity index, normalization of zOTU counts for sequencing depth was performed using the ‘rarefy’ function from the vegan (version 2.6-2) R (version 4.2.0) package (https://www.R-project.org; https://cran.r-project.org/web/packages/vegan). To visualize similarities/differences between bacterial communities, we performed principal coordinate analysis based on Bray–Curtis similarity of square-root-transformed relative abundance of bacterial genera. The differences in the relative abundance of bacterial genera, represented by at least 10 reads in each comparison, were tested using DESeq2. Benjamini-Hochberg corrected *p* values <0.05 were considered significant.

For targeted bacterial measurement using quantitative PCR, bacterial DNA isolated as previously detailed from gut microbiota or from whole-liver DNA extracts isolated using QIAamp Miniprep Kit (QIAgen, Venlo, The Netherlands) was analyzed using quantitative real-time PCR using Power SYBR Green mix/Light-Cycler 480 Detection System (Roche Diagnostics, Rotkreuz, Zug, Switzerland). Targeted bacterial amplification levels were normalized using pan-bacteria primer pairs.

### Statistical analyses

Statistical analyses using one-way ANOVA or Student’s *t* test were performed using GraphPad Prism software (GraphPad Software, San Diego, CA, USA). Bars represent mean ± standard error of the mean (SEM). A *p* value <0.05 was considered statistically significant.

## Results

### Dietary MASLD/MASH mouse models present attenuated expression of intestinal AMPs and *Il18bp* associated with an enrichment in gut proteobacteria

**Fig. 1:**
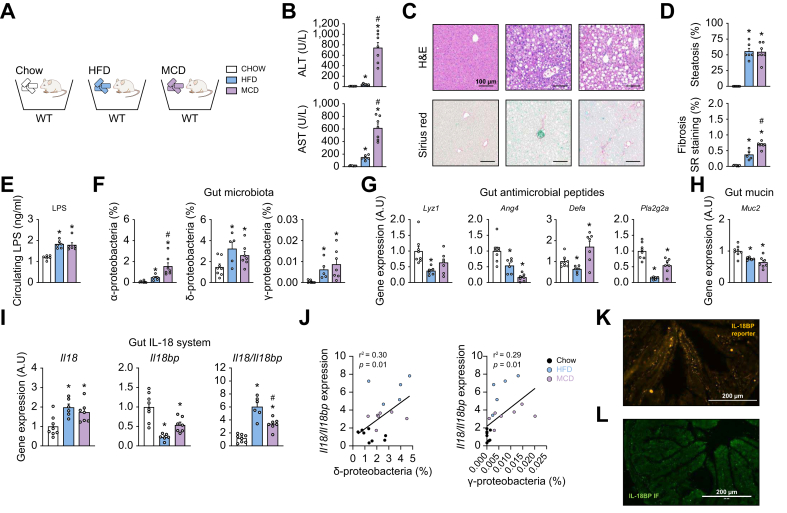
Intestinal antimicrobial peptides (AMPs) and *Il18bp* expressions are decreased while gut proteobacteria are increased in dietary MASLD/MASH mouse models. (A) Schematic representation of the study protocol. (B) Circulating transaminases levels. (C) H&E and Sirius red (SR) staining of liver sections. (D) Liver steatosis evaluation and SR-positive staining quantification. (E) Circulating LPS levels. (F) Proportion of different proteobacteria class in gut microbiota. (G) Ileal gene expression of AMPs. (H) Ileal gene expression of *Muc2*. (I) Ileal gene expression of *Il18*, *Il18bp*, and *Il18*/*Il18bp* ratio. (J) Correlations between *Il18*/*Il18bp* ratio and δ- or γ-proteobacteria content. (K) Ileal immunofluorescence of the IL-18BP tdTomato reporter. (L) Ileal IL-18BP immunostaining in WT mice. Bars represent mean ± SEM of individual values (circles). ∗*p* <0.05 *vs.* chow diet group and ^#^*p* <0.05 *vs.* HFD group (Student's *t* test). n = 5–8 male mice per group. AMPs, antimicrobial peptides; A.U, arbitrary unit; HFD, high-fat diet; MCD, methionine choline deficient; IL-18BP, IL-18-binding protein; LPS, lipopolysaccharide; MASH, metabolic dysfunction-associated steatohepatitis; MASLD, metabolic dysfunction-associated steatotic liver disease; WT, wild-type.

To model MASLD/MASH, C57BL/6J male mice were fed with a HFD for 10 weeks or an MCD diet for 7 weeks (Fig. 1A). As expected, HFD and MCD-diet-fed mice presented elevated circulating transaminases levels (Fig. 1B), and evident histological liver steatosis and fibrosis when compared with control (chow diet)-fed mice (Fig. 1C and D). In further investigating the enterohepatic axis in these two dietary models, we first observed that their blood levels of LPS were significantly increased compared with control mice (Fig. 1E). Accordingly, the different classes of Gram-negative proteobacteria (in particular α-, δ-, and γ-proteobacteria) containing large amounts of LPS in their outer membrane, were enriched in gut microbiota of both HFD and MCD-diet-fed mice (Fig. 1F). As host alterations could explain this proteobacteria excess, we first investigated gut expression of AMPs that are important innate regulators protecting the intestinal epithelium against enteric pathogens. Interestingly, we observed that some important AMPs, such as lysozyme (*Lyz1*), angiogenin 4 (*Ang4*) and phospholipase A2 (*Pla2g2a*) are consistently less expressed in the gut of MASLD/MASH mice when compared with control mice (Fig. 1G). Similarly, intestinal gene expression of *Muc2*, encoding the mucin isoform mainly composing gut mucus, a physiological barrier against pathogens, is decreased in both MASLD/MASH models (Fig. 1H).

Previous works have implicated intestinal IL-18 signaling in the maintenance of gut epithelial integrity in the context of colitis.^8^^,^^9^ Investigating gene expression of members of this pathway, we observed that IL-18 (*Il18*) is overexpressed while its natural blocker IL-18BP (*Il18bp*) is repressed in the ileum of both HFD and MCD-diet-fed mice (Fig. 1I). The *Il18*/*Il18bp* mRNA ratio was in consequence increased in both MASLD/MASH models (Fig. 1I) and positively correlated to the proportion of delta- and gamma-proteobacteria in gut microbiota (Fig. 1J). Gut histological slices of Il18bp-tomato^ki/ki^ (knock-in) reporter mice (harboring endogenous fluorescence in nuclei of IL-18BP-producing cells) (Fig. 1K) and immunofluorescence with anti-IL-18BP antibody (Fig. 1L) identified top-villi enterocytes as the main intestinal epithelial source of IL-18BP.

### IL-18BP deficiency further aggravates AMPs decrease in dietary MASLD/MASH mouse models

**Fig. 2:**
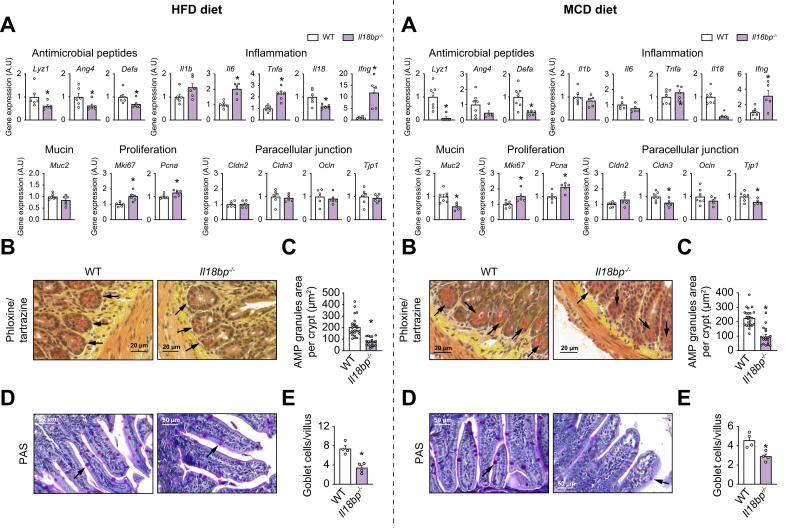
IL-18BP deficiency aggravates AMPs decrease in mice on HFD and MCD diet. Left panels represent results on HFD while right panels represent results on MCD diet. (A) Ileal gene expression of AMPs, cytokines, proliferation markers, and paracellular junction mediators. (B) Histological sections of ileum from *Il18bp*^*-/-*^ and WT mice stained with phloxine–tartrazine. Black arrows show fewer/smaller AMPs granules in Paneth cells from *Il18bp*^*-/-*^ mice *vs.* WT mice. (C) Quantification of AMPs granule area (reported to crypt). (D) Histological sections of ileum from *Il18bp*^*-/-*^ and WT mice stained with PAS. Black arrows indicate localization of some goblet cells. (E) Quantification of goblet cells (reported to villus). Bars represent mean ± SEM of individual values (circles). ∗*p* < 0.05 *vs.* WT mice (Student's *t* test). n = 4–7 male mice per group. AMPs, antimicrobial peptides; HFD, high-fat diet; IL-18BP, IL-18-binding protein; MCD, methionine choline deficient; PAS, periodic acid–Schiff; WT, wild-type.

To better understand the implication of IL-18 signaling balance in intestinal barrier protection, we studied transcriptional regulations in the ileum of *Il18bp*^*-/-*^ mice in MASLD/MASH-inducing conditions (on HFD and MCD diet), and in basal condition (on chow diet). In *Il18bp*^*-/-*^ mice, all IL-18 produced is present as free IL-18, resulting in an unopposed IL-18 signaling.^11^ Gene expression of Paneth cells-derived AMPs (*Lyz1*, *Ang4*, *Defa*) was reduced in *Il18bp*^*-/-*^ *vs.* WT gut mice on all diets (Fig. 2A, Fig. S1). In accordance with these transcriptional observations, Lendrum’s phloxine–tartrazine staining revealed that AMPs secretory granules in Paneth cells, located at the bottom of intestinal crypts, were fewer and smaller in *Il18bp*^*-/-*^ compared with WT mice on both HFD and MCD diet (Fig. 2B and C). Other AMPs (*Reg3b*, *Reg3g*, *Pla2g2a*, *Cramp*) were regulated in the intestine of *Il18bp*^*-/-*^ mice in a diet-dependent manner (Fig. S1). In contrast, ileal gene expression of cell proliferation markers (*Mki67*, *Pcna*) was increased in *Il18bp*^*-/-*^ mice compared with WT mice on HFD and MCD diet (Fig. 2A) but not on chow diet (Fig. S1).

Classical proinflammatory cytokines (*Il6*, *Tnfa*) were overexpressed in the intestine of *Il18bp*^*-/-*^ mice on HFD but not on MCD or chow diets (Fig. 2A, Fig. S1). Some paracellular junction proteins (*Cldn3*, *Tjp1*) and *Muc2* mucin isoform were less expressed in the gut of *Il18bp*^*-/-*^ mice on MCD, but not on HFD or chow diet (Fig. 2A, Fig. S1), while mucus-producing goblet cells per villus were reduced in the ileum of *Il18bp*^*-/-*^ mice on both HFD and MCD diet (Fig. 2D and E). Of note, ileal gene expression of *Il18* was also constitutively decreased while that of *Ifng* was constitutively increased in *Il18bp*^*-/-*^ *vs.* WT mice, whatever the diet considered (Fig. 2A, Fig. S1).

Taken together, these results suggest that intestinal *Il18bp* is repressed during dietary MASLD/MASH in mice and that its deficiency lowers gut AMPs production.

### IL-18BP deficiency constitutively alters gut microbiota in a diet-amplified manner

**Fig. 3:**
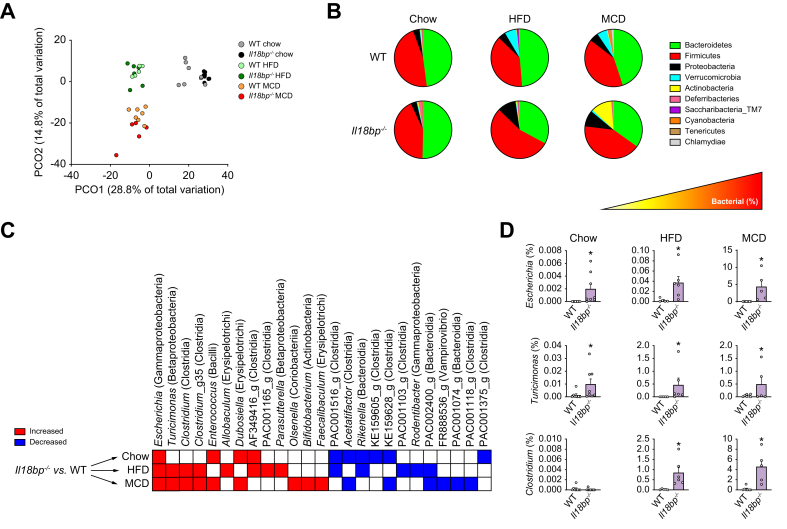
IL-18BP deficiency alters gut microbiota composition in a diet-amplified manner. (A) Principal component analysis of gut microbiota. (B) Proportion of phyla (pie chart) composing gut microbiota. (C) List of bacterial genera differentially regulated between genotypes within diets. (D) Quantification of bacterial genera changes between genotypes on different diets. Note the different scales on Y-axes between diets. Bars represent mean ± SEM of individual values (circles). ∗*p* <0.05 *vs.* WT mice (Student's *t* test). n = 5–8 male mice per group. HFD, high-fat diet; IL-18BP, IL-18-binding protein; MCD, methionine choline deficient; WT, wild-type.

As AMPs interfere with gut microbiome architecture, we analyzed (through next generation sequencing), gut microbiota composition of *Il18bp*^*-/-*^ and WT mice in basal conditions (chow diet) and under HFD and MCD diet. Principal component analysis (PCA) based on the bacterial genera proportion (Fig. 3A) clustered *Il18bp*^*-/-*^ and WT mice within and across diets, indicating a genotype–diet interaction. Significant differences in gut microbiota composition were already observed at the phylum levels within and across diets (Fig. 3B). Interestingly, *Il18bp*^*-/-*^ mice exhibited a consistent increase in β- and γ-proteobacteria on diets inducing MASLD/MASH (Fig. S2). A deeper taxonomic analysis identified genera (in particular *Escherichia*, *Clostridium*, and *Turicimonas*) similarly upregulated in *Il18bp*^*-/-*^ *vs.* WT mice across diets (Fig. 3C). Of note, a successive 10-fold enrichment in *Escherichia* proportion was observed in *Il18bp*^*-/-*^ mice across diets (Chow < HFD < MCD, as shown by the different scales on Y-axes) (Fig. 3D). Taken together, these results suggest that defects in AMPs production observed in *Il18bp*^*-/-*^ mice are associated with an altered gut microbiota composition in this mouse line.

### IL-18BP deficiency exacerbates hepatic inflammation and fibrosis in mice on MASLD/MASH-inducing diets

**Fig. 4:**
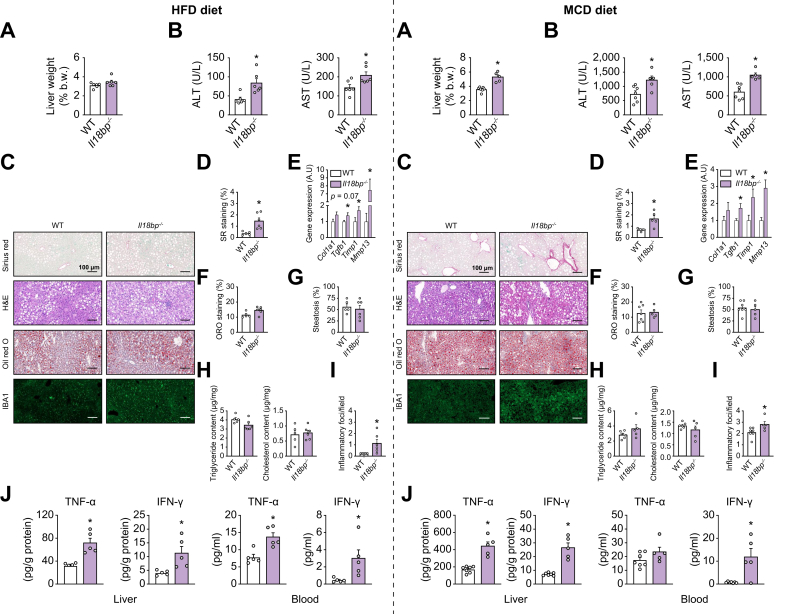
IL-18BP deficiency worsens hepatic inflammation and fibrosis in mice on HFD and MCD diet. Left panels represent results on HFD while right panels represent results on MCD diet. (A) Relative liver weight. (B) Circulating transaminases levels. (C) Sirius red (SR), H&E, Oil red O (ORO) and IBA1 staining of liver sections. (D) SR-positive staining quantification. (E) Liver gene expression of pro-fibrogenic markers. (F) ORO-positive staining quantification. (G) Liver steatosis evaluation. (H) Liver triglyceride and cholesterol content. (I) Number of inflammatory foci per field (200 × ). (J) Liver and blood cytokine protein content. Bars represent mean ± SEM of individual values (circles). ∗*p* <0.05 *vs.* WT mice (Student's *t* test). n = 5–7 male mice per group. HFD, high-fat diet; IBA1, ionized calcium–binding adapter molecule 1; IL-18BP, IL-18-binding protein; MCD, methionine choline deficient; WT, wild-type.

To verify if defects in AMPs production and gut microbiota composition induced by IL-18BP deficiency could affect whole-body physiology, we extended our phenotyping of *Il18bp*^*-/-*^ mice. In basal conditions (chow diet), *Il18bp*^*-/-*^ mice exhibit similar body weight, adiposity, and liver status compared to WT mice (Fig. S3), corroborating the absence of spontaneous immune phenotype previously reported in this mouse model.^11^ On HFD, body weight, body composition, glucose tolerance and insulin sensitivity, oxygen consumption, and respiratory exchange ratio remain similar in *Il18bp*^*-/-*^ and WT mice (Fig. S4), ruling out an impact of IL-18BP deficiency on systemic glucose and energy homeostasis. In this metabolic context, to investigate the impact of IL-18BP deficiency on MASLD/MASH severity, we compared liver phenotype of *Il18bp*^*-/-*^ *vs.* WT mice on HFD (Fig. 4 left panels) and MCD diet (Fig. 4 right panels). *Il18bp*^*-/-*^ mice relative liver weight was similar on HFD but increased on MCD diet (Fig. 4A). Interestingly, on both diets, *Il18bp*^*-/-*^ mice exhibited an increase in circulating transaminases levels (Fig. 4B) and initiation of liver fibrosis, occurring independently of major changes in liver steatosis (Fig. 4C–H). Inflammatory foci number and protein content in the proinflammatory cytokines tumor necrosis factor-alpha (TNF-α) and IFN-γ were increased in the liver of diet-challenged *Il18bp*^*-/-*^ *vs.* WT mice (Fig. 4I and J). To better characterize the over-inflammation observed in *Il18bp*^*-/-*^ mice, we performed RNA-seq analysis (Fig. S5A), as well as a wide screening of gene expression through qPCR (Fig. S5B). Differential gene expression analysis and Gene Set Enrichment Analysis confirmed that transcripts significantly upregulated in *Il18bp*^*-/-*^ mice livers were enriched in gene ontology clusters related to inflammation (Fig. S5A). Compared with WT mice, *Il18bp*^*-/-*^ mice on HFD and MCD diet exhibited hepatic upregulation of macrophages markers (Kupffer cells, blood-derived macrophages, M1-activated macrophages) and dendritic cells (Fig. S5B). Downstream targets of IFN-γ (*Ciita*, *Cxcl9*, *Cxcl10*) were also overexpressed in the liver of *Il18bp*^*-/-*^ mice on both diets, while transcriptional upregulation for other proinflammatory cytokines (*Il1b*, *Il6*, *Il12*) appeared diet-specific in *Il18bp*^*-/-*^ mice (Fig. S5B).

### Altered gut microbiota resulting from IL-18BP deficiency primes liver over-inflammation on MASLD/MASH-inducing diets

**Fig. 5:**
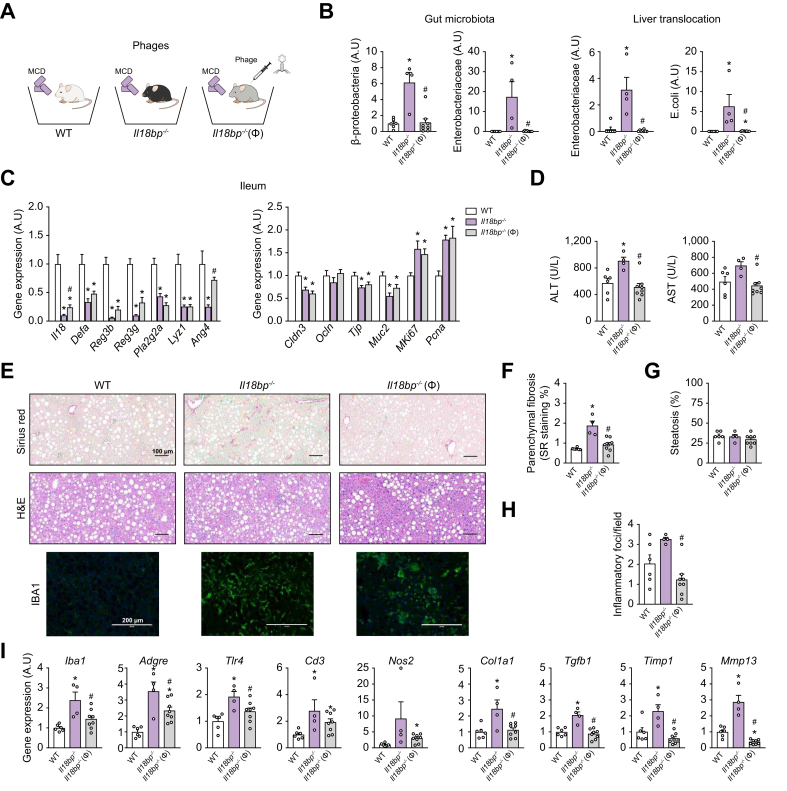
Contribution of specific gut microbiota to hepatic over-inflammation/fibrosis in IL-18BP deficiency (phages administration to *Il18bp*^*-/-*^ mice). (A) Schematic representation of the study protocol. (B) Quantification of bacterial genera changes in gut and bacteria translocation in liver. (C) Ileal gene expression of *Il18*, antimicrobial peptides, paracellular diffusion mediators and proliferation markers. (D) Circulating transaminases levels. (E) Sirius red (SR), H&E and IBA1 staining of liver sections. (F) SR-positive staining quantification. (G) Liver steatosis evaluation. (H) Number of inflammatory foci per field (200 × ). (I) Liver gene expression of pro-fibrogenic and immune/inflammatory markers. Bars represent mean ± SEM of individual values (circles). All mice were fed with MCD diet amplifying differences in gut microbiota between genotypes. ∗*p* <0.05 *vs.* WT mice and ^#^*p* <0.05 *vs. Il18bp*^*-/-*^ untreated mice (Student's *t* test). n = 4–8 male mice per group. ALT, alanine aminotransferase; AST, aspartate aminotransferase; IBA1, ionized calcium–binding adapter molecule 1; IL-18BP, IL-18-binding protein; MCD, methionine choline deficient; WT, wild-type.

**Fig. 6:**
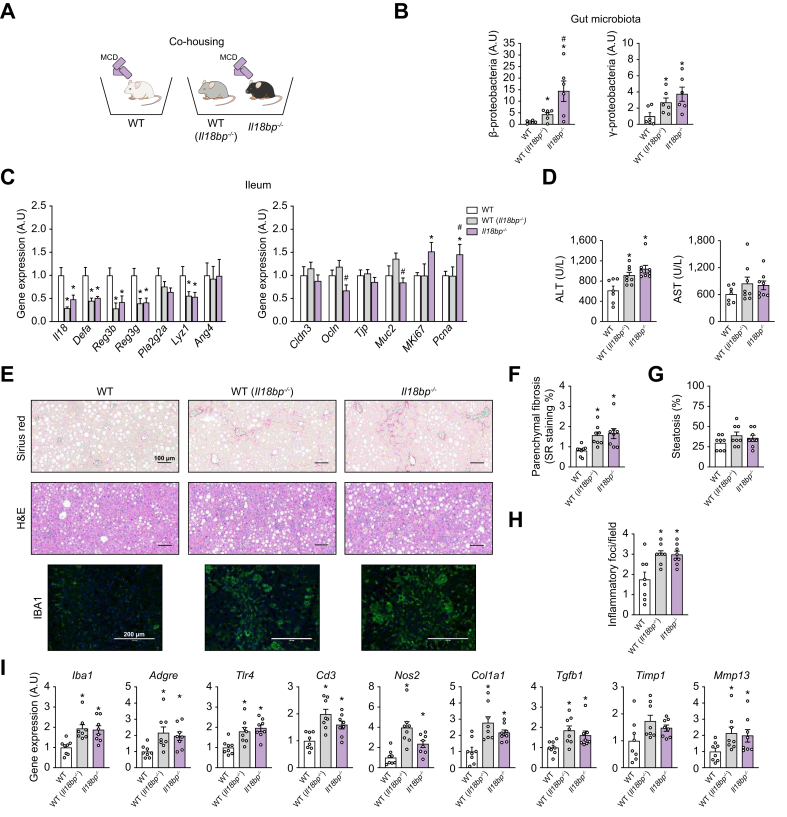
Contribution of gut microbiota to hepatic over-inflammation/fibrosis in IL-18BP deficiency (co-housing of WT with *Il18bp*^*-/-*^ mice). (A) Schematic representation of the study protocol. (B) Quantification of bacterial genera changes in gut. (C) Ileal gene expression of *Il18*, antimicrobial peptides, paracellular diffusion mediators and proliferation markers. (D) Circulating transaminases levels. (E) Sirius red (SR), H&E, and IBA1 staining of liver sections. (F) SR-positive staining quantification. (G) Liver steatosis evaluation. (H) Number of inflammatory foci per field (200 × ). (I) Liver gene expression of pro-fibrogenic and immune/inflammatory markers. Bars represent mean ± SEM of individual values (circles). All mice were fed with MCD diet amplifying differences in gut microbiota between genotypes. ∗*p* <0.05 *vs.* WT mice and ^#^*p* <0.05 *vs.* WT mice co-housed with *Il18bp*^*-/-*^ mice (WT[*Il18bp*^*-/-*^]) (Student's *t* test). n = 6–8 male mice per group. ALT, alanine aminotransferase; AST, aspartate aminotransferase; IBA1, ionized calcium–binding adapter molecule 1; IL-18BP, IL-18-binding protein; MCD, methionine choline deficient; WT, wild-type.

To investigate whether alterations in AMPs production and gut microbiota composition observed in *Il18bp*^*-/-*^ mice were involved in worsened hepatic inflammatory/fibrotic status, we subjected *Il18bp*^*-/-*^ mice to (i) oral administration of a phages solution directed against enteropathogenic bacteria (Fig. 5), (ii) oral administration of a broad*-*spectrum antibiotic (Fig. S7), or iii) co-housing with WT mice, allowing sharing of microbial communities between genotypes (Fig. 6). First, we initiated these experimental interventions on MCD diet, which amplifies gut microbiota differences between *Il18bp*^*-/-*^ and WT mice (Fig. 3).

To target the Enterobacteriaceae over-represented in gut microbiota of *Il18bp*^*-/-*^ mice, we used a commercially-validated *E. coli*-*Proteus* bacteriophage solution.^14^^,^^15^ Quantitative PCR allowed us to validate *in vitro* the susceptibility of *E. coli* isolate from *Il18bp*^*-/-*^ mice microbiota to this phage preparation (Fig. S6).

Oral administration of phages lysates (an emerging antimicrobial strategy to target the gut–liver axis^16^) to *Il18bp*^*-/-*^ mice led to a massive reduction in β-proteobacteria and Enterobacteriaceae in their gut microbiota (Fig. 5B). In addition, the presence of Enterobacteriaceae*/E. coli* DNA, which was only detected in the liver of *Il18bp*^*-/-*^ mice (not in the liver of WT mice), was suppressed in those of phages-treated *Il18bp*^*-/-*^ mice (Fig. 5B). At the intestinal level, phages administration led to an increase in *Ang4* mRNA and a more modest upregulation of *Il18* expression in *Il18bp*^*-/-*^ mice (Fig. 5C). The expression of *Reg3b* and *Reg3g* mRNA also showed a trend to increase in phages-treated *vs*. untreated *Il18bp*^*-/-*^ mice ileum. In contrast, the expression of other AMPs (*Defa*, *Pla2g2a*, *Lyz1*) and mediators of paracellular diffusion (*Cldn3*, *Tjp*), all decreased in *Il18bp*^*-/-*^ mice ileum, were not affected by phages treatment (Fig. 5C). Similarly, markers of cell proliferation (*MKi67*, *Pcna*), which were increased in *Il18bp*^*-/-*^ mice ileum, were not changed by phages treatment (Fig. 5C). At the liver level, phages administration was associated with a mitigation of hepatic damages (reflected by reduced blood transaminases levels (Fig. 5D), inflammatory and fibrotic markers) (Fig. 5E–I) in *Il18bp*^*-/-*^ mice. These observations evidence that targeted eradication of pathogenic gut bacterial strains limits diet-induced over-inflammation observed in *Il18bp*^*-/-*^ mice livers.

Similarly, oral administration of ampicillin resulted in a drastic reduction in gut bacterial content and ileal AMPs expression in both WT and *Il18bp*^*-/-*^ mice (Fig. S7B and C), suppressing their difference in terms of liver phenotype (Fig. S7D–I). This suggests that gross eradication of gut microbiota also limited susceptibility to hepatic over-inflammation/damage in *Il18bp*^*-/-*^ mice.

Finally, WT mice co-housed with *Il18bp*^*-/-*^ mice (named WT [*Il18bp*^*-/-*^]) exhibited a rise in β- and γ-proteobacteria in their gut microbiota when compared with non-co-housed WT mice (Fig. 6B). This microbial enrichment was associated with an intestinal downregulation of AMPs (*Defa*, *Reg3b*, *Reg3g*, *Lyz1*) in WT (*Il18bp*^*-/-*^), mimicking that of *Il18bp*^*-/-*^ mice (Fig. 6C). In contrast, WT (*Il18bp*^*-/-*^) exhibited no change in gene expression of mediators of paracellular diffusion and markers of cell proliferation when compared with non-co-housed WT mice (Fig. 6C). Interestingly, WT (*Il18bp*^*-/-*^) showed similar liver damages as those of *Il18bp*^*-/-*^ mice, when compared with non-co-housed WT mice. This included elevation of circulating ALT levels (Fig. 6D) and hepatic fibrosis and inflammatory foci content (Fig. 6E–H), associated with an overexpression of proinflammatory and pro-fibrotic marker genes (Fig. 6I). Together, these results suggest a transmissibility of hepatic over-inflammation/fibrosis seen in *Il18bp*^*-/-*^ mice.

Repeating these different experimental interventions on HFD, we observed that several of the hepatic improvements triggered by phages administration and antibiotic treatment in *Il18bp*^*-/-*^ mice on MCD diet were also visible on HFD (Figs. S8 and S9). Similarly, WT [*Il18bp*^*-/*^^*-*^]) also presented worsened circulating ALT levels and hepatic inflammatory/pro-fibrogenic transcripts levels when compared with non-co-housed WT mice on HFD (Fig. S10).

## Discussion

Clinical investigation of gut–liver axis in MASLD/MASH patients remains challenging, due to invasiveness of liver/gut biopsies and the slow rate of disease progression. In this context, animal models are particularly useful to elucidate underlying pathophysiological mechanisms. HFD and MCD diet are classical diets used to model MASLD/MASH in mice. Hypercaloric HFD simulates the human metabolic syndrome but requires prolonged consumption to result in hepatic damage including limited fibrosis. In contrast, MCD diet induces MASLD/MASH more rapidly/efficiently through the inhibition of hepatic fatty acid oxidation and very low-density lipoprotein (VLDL) synthesis, but its non-physiological composition does not fully mimic the human situation.^17^

In the present study, we first noticed that WT mice on HFD and MCD diet exhibit a decreased intestinal expression of AMPs and mucin while their gut microbiota was enriched in Gram-negative LPS-rich proteobacteria. These results corroborate previous basic studies highlighting gut–liver axis dysregulation in diet models of MASLD/MASH in mice,[18], [19], [20] as well as more recent work reporting reduction of AMPs expression in the ileum of cirrhotic patients.^21^

Interestingly, we observed that gene expression of IL-18 was increased while that of IL-18BP was decreased in ileum of HFD and MCD diet-fed mice. In consequence, the intestinal IL-18/IL-18BP mRNA ratio was elevated in both MASLD/MASH models, positively correlating with the proportion of proteobacteria families present in their gut microbiota. Functionally, IL-18BP forms high affinity complexes with IL-18, thus blocking its interaction with IL-18 receptors.^3^^,^^4^ Moreover, the binding affinity of IL-18 with IL-18BP is much higher than to its IL-18 receptor and the abundant concentrations of IL-18BP leaves very few free and bioactive IL-18 molecules in basal condition.^3^^,^^4^

To mechanistically understand the role of intestinal IL-18/IL-18BP balance in the host–microbiota interaction, we studied transcriptional and histological changes in the ileum of mice genetically deficient in IL-18BP (*Il18bp*^*-/-*^ mice) placed on HFD or MCD diet. In *Il18bp*^*-/-*^ mice, all IL-18 produced is free and thus bioactive, leading to an ‘unopposed’ IL-18 signaling.^11^ *Il18bp*^*-/-*^ mice present a decreased expression of Paneth cells-derived AMPs (*Lyz1*, *Ang4*, *Defa*) associated with a decrease in AMPs secretory granules content. In addition, *Il18bp*^*-/-*^ mice present a reduction in mucus-producing goblet cells in their ileal villi on both HFD and MCD diets, in line with the loss of goblet cells maturation previously reported in the colon of *Il18bp*^-/-^ mice treated with dextran sodium sulfate.^9^ In line with these defects in host defenses, *Il18bp*^*-/-*^ mice exhibited marked changes in gut microbiota composition, which are importantly amplified on HFD and MCD diet. In fact, *Il18bp*^*-/-*^ mice exhibited a consistent increase in β- and γ-proteobacteria (in particular *Escherichia* and *Turicimonas*), as well as in *Clostridium*, across diets. From a translational point of view, an enrichment in the same bacterial genera has been recently observed in gut microbiota of patients affected by MASLD/MASH.[22], [23], [24], [25] In addition, a large clinical study has shown that long-term gut microbiome instability with dominance of Enterobacteriaceae and *Escherichia*/*Shigella* correlated with the development of MASLD in humans.^26^ Taken together, these results confirm that IL-18BP deficiency results in gut microbiota modifications that are also present in patients with MASLD/MASH.

Previous works have implicated IL-18 in the homeostatic crosstalk between the intestinal epithelium and gut bacteria, in particular in the context of colitis and inflammatory bowel disease. Levy *et al.*^8^ described that epithelial IL-18 activates AMPs production via nuclear factor kappa B-dependent signaling to regulate microbial community and prevent intestinal inflammation. Chiang *et al.*^27^ reported that basal IL-18 signaling contributes to Stat3-mediated antimicrobial response in Paneth cells. Functionally, administration of IL-18 can prevents colonization of proinflammatory bacteria in pathogen/germ-free *IL10*^-/-^ mice^28^ and ameliorates the clearance of adherent-invasive *E. coli* in *IL22*^-/-^ mice.^27^ Conversely, mice genetically deficient in IL-18 (*Il18*^*-/-*^ mice) present increases in *Prevotellaceae* and the TM7 phylum that are responsible for release of Toll-like receptor (TLR) agonists in the portal circulation.^10^ Our results add another layer of complexity to the understanding of the role of IL-18 signaling in AMP regulation and the host–microbiota dialog. Indeed, we evidenced that control of IL-18 signaling by IL-18BP is just as necessary as the presence of IL-18 itself to ensure the required production of AMP needed to avoid instauration of MASH-associated microbiome.

In addition, we also observed that WT mice co-housed with *Il18bp*^*-/-*^ mice (thus sharing with them an elevated proportion in β- and γ-proteobacteria in gut microbiota) also exhibit reduced AMPs expression in the ileum, while treatment with phages directed against Enterobacteriaceae tend toward limiting AMPs repression in *Il18bp*^*-/-*^ mice ileum. These results suggest that pathogenic γ-proteobacteria/Enterobacteriaceae could contribute to negatively regulate AMPs expression. These observations complete previous ones showing that commensal gut microbiota and its byproducts stimulate gut IL-18 production and AMPs program. In fact, intestinal IL-18 levels progressively increase postnatally, paralleling microbial colonization,^29^ and germ-free mice colonized with human gut microbiota exhibit a transient increase in IL-18.^30^ Mechanistically, some bacterial metabolites such as taurine or polyamines can activate the inflammasome-dependent production of IL-18 that primes AMPs production.^8^ Altogether, these data show that if host IL-18 signaling drives production of AMPs to regulate gut microbiota, in turn, some bacteria could positively or negatively regulate IL-18 amount, closing a homeostatic loop of intestine–gut microbiota crosstalk.

Gut inflammation and intestinal barrier defects are other biological mechanisms which can accelerate MASLD/MASH.^31^ We observed a concomitant decrease of *Il18* and increase of *Ifng* mRNAs content in the intestine of *Il18bp*^*-/-*^ mice on all diets, reflecting both negative feedback on IL-18 transcription and constant stimulation of IFN-γ production in response to the unopposed IL-18 signaling. Elevated levels of ileal IFN-γ expression appeared not involved in reduction of AMPs in *Il18bp*^*-/-*^ mice. In fact, no difference in terms of ileal AMPs expression can be observed between *Il18bp*^*-/-*^ mice and the double KO *Il18bp*^*-/-*^ *Ifng*^*-/-*^ mouse line (data not shown). In contrast, other proinflammatory cytokines (*Il6*, *Tnfa*) are only overexpressed in the ileum of *Il18bp*^*-/-*^ mice on HFD, not on MCD and chow diets, suggesting no constitutive intestinal inflammation in *Il18bp*^*-/-*^ mice, as previously reported at the systemic level.^11^ Several studies have highlighted a dual role for IL-18 signaling in inflammation of the intestinal epithelium. In mice, systemic administration of IL-18 induced intestinal mucosal inflammation,^32^ while administration of IL-18BP reduced intestinal inflammation and ulceration.^33^ Genetic deletion of IL-18 or its receptor IL-18R1 in intestinal epithelial cells protected mice from chemically induced mucosal inflammation.^9^ In humans, IL-18 is produced by gut epithelial cells and macrophages and this production is increased during inflammatory bowel diseases[34], [35], [36], [37] validating the use of anti-IL-18 monoclonal antibody for Crohn’s disease (ongoing clinical trial NCT03681067). Nevertheless, more recent works highlight a protective role for physiological amount of IL-18 on intestinal inflammation, in particular through its crosstalk with IL-22.^27^^,^^38^ In addition, IL-18 polymorphisms reducing IL-18 levels have been implicated in susceptibility to Crohn’s disease.^39^ It has also been shown that epithelium-derived IL-18 stimulates epithelial proliferation through induction of stem cell genes,^27^ in accordance with increased ileal expression of cell proliferation markers (*Mki67*, *Pcna*) we observed in *Il18bp*^*-/-*^ mice on HFD and MCD diet.

Altogether, these observations reveal that as for AMPs regulation, the control of inflammation and renewal of the epithelium require a fine-tuning of IL-18 signaling in which its binding protein IL-18BP plays a central role.

Finally, *Il18bp*^*-/-*^ mice present an aggravation of liver damage, in particular regarding inflammation and fibrosis, when compared to diet-matched WT mice on HFD and MCD diet. Livers from HFD and MCD diet-fed *Il18bp*^*-/-*^ mice exhibit enrichment in markers of immune cell types (monocyte-derived macrophages, myeloid dendritic cells and T-cells) that have been involved in innate and adaptative immune responses to MASH progression.^40^ Of note, this propensity to hepatic dietary over-inflammation reflects neither a constitutive proinflammatory state (liver from *Il18bp*^*-/-*^ and WT mice being similar on chow diet) nor the liver manifestation of a worsened metabolic syndrome (energy and glucose homeostasis remaining similar in *Il18bp*^*-/-*^ and WT mice on HFD). In contrast, our phages and co-housing experiments demonstrate that altered gut microbiota of *Il18bp*^*-/-*^ mice is sufficient to trigger aggravation of MASLD/MASH. Moreover, we detected the presence of *Enterobacteriaceae* DNA in the liver of *Il18bp*^*-/-*^ mice, while it was undetectable in that of WT mice. We also observed hepatic overexpression of TLRs) (*Tlr4* and *Tlr9*) in both HFD and MCD-fed *Il18bp*^*-/-*^ mice. Endotoxins of the Gram-negative bacterial wall acts as major pathogen-associated molecular patterns for TLRs.^41^ In the liver, endotoxin such as LPS powerfully stimulates TLR4 in Kupffer cells, which, in turn, activates overproduction of proinflammatory cytokines such as TNF-α and IL-6.^42^ In addition, gut microbiota-derived LPS contribute to the progression of liver fibrosis.^43^ Taken together, these observations demonstrate the primary role of the pathogenic intestinal microbiota of *Il18bp*^*-/-*^ mice in their worsened steatohepatitis. Nevertheless, further work involving intestine-specific and hematopoietic-specific/*Il18bp*^*-/-*^ mice would be useful to better evaluate possible additional intrahepatic contribution of IL-18BP in the protection against MASLD/MASH progression.

In conclusion, IL-18BP deficiency in mice alters AMPs production and leads to gut dysbiosis that shares similarities with gut microbiota signature of MASLD/MASH patients. These results implicate IL-18BP, as well as IL-18, in the physiological homeostasis of gut–liver axis. Increasing IL-18BP amount/activity (a therapeutic option already clinically validated to treat a rare systemic auto-inflammatory disease)^44^ could represent an interesting therapeutic perspective to treat MASLD/MASH as well as gut microbiota dysbiosis.

## Abbreviations

ALT, alanine aminotransferase; AMP, antimicrobial peptide; AST, aspartate aminotransferase; A.U, arbitrary unit; GTT, glucose tolerance test; HCC, hepatocellular carcinoma; HFD, high-fat diet; IBA1, ionized calcium–binding adapter molecule 1; IFN-γ, interferon-gamma; IL-18BP, IL-18-binding protein; IL-18R, interleukin-18 receptor; ITT, insulin tolerance test; LPS, lipopolysaccharide; MASH, metabolic dysfunction-associated steatohepatitis; MASLD, metabolic dysfunction-associated steatotic liver disease; MCD, methionine choline deficient; ORO, Oil Red O; PAS, periodic acid–Schiff; PCA, principal component analysis; SR, Sirius Red; TLR, Toll-like receptor; TNF-α, tumor necrosis factor-alpha; WT, wild-type; zOTUs, zero-radius operational taxonomic units.

## Financial support

This work was funded by the 10.13039/501100001711SNSF grant number 215330 (IL-18 signaling in NAFLD/NASH: from mice to humans), the Foundation of the Swiss Diabetes Association, the Swisslife Foundation, the Vontobel stiftung, and the Novartis stiftung. We thank Prof. Jacques Phillippe for his kind support.

## Authors’ contributions

Designed the study: ES, CG, FRJ. Performed the experiments: ES, EP, KL, FI, SAM, CVD, FB, FV, VL. Performed RNA-seq bioinformatics analyses: YJ, DR. Performed gut microbiota analyses: NG, VL. Interpret results throughout the study: ES, VL, ACG, JS, DR, KG, CG, FRJ. Provided material and methods: ACG. Wrote the manuscript: ES. Participated in data interpretation, revision, and editing of the manuscript: all authors.

## Data availability

Raw data supporting the findings of this study are available on request from the corresponding authors.

## Conflicts of interest

The authors declare no conflicts of interest.

Please refer to the accompanying ICMJE disclosure forms for further details.
