# Supplementary material for: Interleukin-18 binding protein deficiency results in gut microbiota dysbiosis and aggravated diet-induced MASH in mice
Source: JHEP Rep. 2025 Oct 10;8(1):101629. doi: 10.1016/j.jhepr.2025.101629 (PMC12753522; doi:10.1016/j.jhepr.2025.101629)
Supplement: Multimedia component 1 [file mmc1.pdf]

Interleukin-18 binding protein deficiency results in gut microbiota dysbiosis and aggravated diet-induced MASH in mice

Emmanuel Somm, Elodie Perroud, Yunju Jo, Karina Lindner, Frédérique Ino, Sophie A. Montandon, Christelle Veyrat-Durebex, Franck Bontems, Florian Visentin, Nadia Gaïa, Vladimir Lazarevic, Anne-Claude Gavin, Jacques Schrenzel, Dongryeol Ryu, Karim Gariani, Cem Gabay, François R. Jornayvaz

Table of contents

Supplementary figures legends....................2
Fig. S1....................5
Fig. S2....................6
Fig. S3....................7
Fig. S4....................8
Fig. S5....................9
Fig. S6....................10
Fig. S7....................11
Fig. S8....................12
Fig. S9....................13
Fig. S10....................14

Fig. S1. Intestinal transcriptional changes in IL-18BP deficient mice on different diets.

Ileal gene expression of antimicrobial peptides (AMPs), cytokines, proliferation markers, mucin and paracellular junction mediators assessed by qPCR. Bars represent mean ± SEM of individual values (circles). *$p < 0.05$ vs. WT mice (Student's t-test). n = 5-8 male mice per group.

Fig. S2. IL-18BP deficiency alters diet-dependently gut microbiota composition

Gut microbiota analysis in mice on chow diet (A), high-fat diet (HFD) (B) and methionine and choline deficient (MCD) diet (C). For each nutritional condition, the Shannon diversity index and the quantification of bacterial class changes are represented. Bars represent mean ± SEM of individual values (circles). Levels of significance: *$p < 0.05$ vs. WT mice (*Student's t-test*). n = 5-10 male mice per group.

Fig. S3. IL-18BP deficiency does not induce metabolic or liver modifications in basal conditions (chow diet)

(A) Body weight curve. (B) Daily food intake. (C) Body fat proportion (expressed as percentage of body weight). (D) Relative liver weight (expressed as percentage of body weight). (E) Circulating transaminases levels. (F) Haematoxylin/eosin (*H&E)* staining of liver sections. (G) Insulin tolerance test. (H) Liver gene expression of lipid enzymes and transporters. (I) Liver gene expression of immune and inflammatory markers. (J) Liver gene expression of pro-fibrogenic markers. (K) Circulating triglycerides levels. (L) Circulating total cholesterol levels. (M) Circulating high-density lipoprotein (HDL) and low-density lipoprotein (LDL) cholesterol levels. Bars represent mean ± SEM of individual values (circles). Levels of significance: *$p < 0.05$ vs. wild-type (WT) mice (*Student's t-test*). n = 7–8 male mice per group.

Fig. S4. IL-18BP deficiency alters neither glucose homeostasis nor energy homeostasis in mice on HFD

(A) Body weight curve and body fat proportion (as % of body weight) (insert panel). (B) Glucose tolerance test (GTT). (C) Insulin tolerance test (ITT). (D) VO_2 (ml/kg$^{0.75}$/h) curves during light and dark phases. (E) VO_2 (ml/kg$^{0.75}$/h) means during light and dark phases. (F) Respiratory exchange ratio (RER) (= VCO_2/VO_2) curves during light and dark phases. (G) RER means during light and dark phases. (A-C) Each point represents mean ± SE of individual values. (E and G) Bars represent mean ± SEM of individual values (circles). n = 6-12 male mice per group.

Fig. S5. Transcriptomic analysis reveals worsened hepatic inflammation in IL-18BP deficient mice on high-fat diet (HFD) and methionine-choline deficient (MCD) diet

(A) RNAseq analysis including volcano plots, enrichment plots, annotated heat map and pathway networks recapitulating Gene Set Enrichment Analysis (GSEA). (B) Liver gene expression of inflammatory and immune markers assessed by qPCR. DCs: dendritic cells, M1: Classically activated macrophages, M2: Alternatively activated macrophages. Bars represent mean ± SEM of individual values (circles). *$p < 0.05$ vs. WT mice (Student's t-test). n = 4-5 male mice per group (A); n = 5-7 male mice per group (B).

Fig. S6. In vitro validation of the susceptibility of E. coli isolates from *Il18bp$^{-/-}$* mice microbiota to E. coli-Proteus bacteriophage solution

(A) Photography of representative phages sensitive and phages resistant bacterial colonies isolates from gut microbiota of *Il18bp$^{-/-}$* mice.

B) Amplification curve of bacterial DNA extracted from phages sensitive and phages resistant colonies of bacteria. All bacterial colonies were negative in qPCR for clostridium (negative control) while all colonies showed a high genic amplification with the pan-bacteria primers (positive control/normalization). Bacterial colonies sensitive to phages were all Enterobacteriaceae and E. coli positive, while bacterial colonies resistant to phages were all Enterobacteriaceae and E. coli negative.

Fig. S7. Antibiotic administration limits hepatic over-inflammation in IL-18BP deficiency (on MCD diet)

(A) Schematic representation of the study protocol. (B) Quantification of global gut bacterial amount. (C) Ileal gene expression of *Il18* and antimicrobial peptides (AMPs). (D) Sirius red (SR), Haematoxylin/eosin (H&E) and IBA1 staining of liver sections. (E) Circulating transaminases levels. (F) SR positive staining quantification. (G) Liver steatosis evaluation. (H) Number of inflammatory foci per field (200x). (I) Liver gene expression of immune/inflammatory and pro-fibrogenic markers. Bars represent mean ± SEM of individual values (circles). WT and *Il18bp$^{-/-}$* mice treated with antibiotic (ampicillin (1g/L) in drinking water) were compared to untreated WT mice. All mice were fed MCD diet. *$p < 0.05$ vs. WT untreated mice and $^{\#}p < 0.05$ vs. WT antibiotic-treated mice (WT(Abx)) (*Student's t-test*). n = 4-7 male mice per group.

Fig. S8. Administration of phages directed against enteropathogenic bacteria to WT and IL-18BP deficient mice on HFD

(A) Schematic representation of the study protocol. (B) Circulating transaminases levels. (C) Sirius red (SR) and Haematoxylin/eosin (H&E) staining of liver sections. (D) Liver

steatosis evaluation. (E) Number of inflammatory foci per field (200x). (F) Liver gene expression of immune and pro-fibrogenic markers. Bars represent mean ± SEM of individual values (circles). *p < 0.05 for *Il18bp*$^{-/-}$ vs. respective WT mice of the same condition (genotype effect) (*Student's t-test*); $^{\#}p$ < 0.05 vs. WT control mice (phages effect on WT) (*Student's t-test*); $^{\$}p$ < 0.05 vs. *Il18bp*$^{-/-}$ control mice (phages effect on *Il18bp*$^{-/-}$ mice) (*Student's t-test*). n = 6–8 male mice per group.

Fig. S9. Antibiotic administration to WT and IL-18BP deficient mice on HFD

(A) Schematic representation of the study protocol. (B) Circulating transaminases levels. (C) Sirius red (SR) and Haematoxylin/eosin (H&E) staining of liver sections. (D) Liver steatosis evaluation. (E) Number of inflammatory foci per field (200x). (F) Liver gene expression of immune and pro-fibrogenic markers. Bars represent mean ± SEM of individual values (circles). *p < 0.05 for *Il18bp*$^{-/-}$ vs. respective WT mice of the same condition (genotype effect) (*Student's t-test*); $^{\#}p$ < 0.05 vs. WT control mice (antibiotic effect on WT) (*Student's t-test*); $^{\$}p$ < 0.05 vs. *Il18bp*$^{-/-}$ control mice (antibiotic effect on *Il18bp*$^{-/-}$ mice) (*Student's t-test*). n = 6 male mice per group.

Fig. S10. Co-housing of WT mice with IL-18BP deficient mice on HFD

(A) Schematic representation of the study protocol. (B) Circulating transaminases levels. (C) Sirius red (SR) and Haematoxylin/eosin (H&E) staining of liver sections. (D) Liver steatosis evaluation. (E) Number of inflammatory foci per field (200x). (F) Liver gene expression of immune and pro-fibrogenic markers. Bars represent mean ± SEM of individual values (circles). *p < 0.05 vs. isolated WT mice (*Student's t-test*); $^{\#}p$ < 0.05 vs. WT mice co-housed with *Il18bp*$^{-/-}$ mice (WT(*Il18bp*$^{-/-}$)) (*Student's t-test*). n = 8 male mice per group.

S1

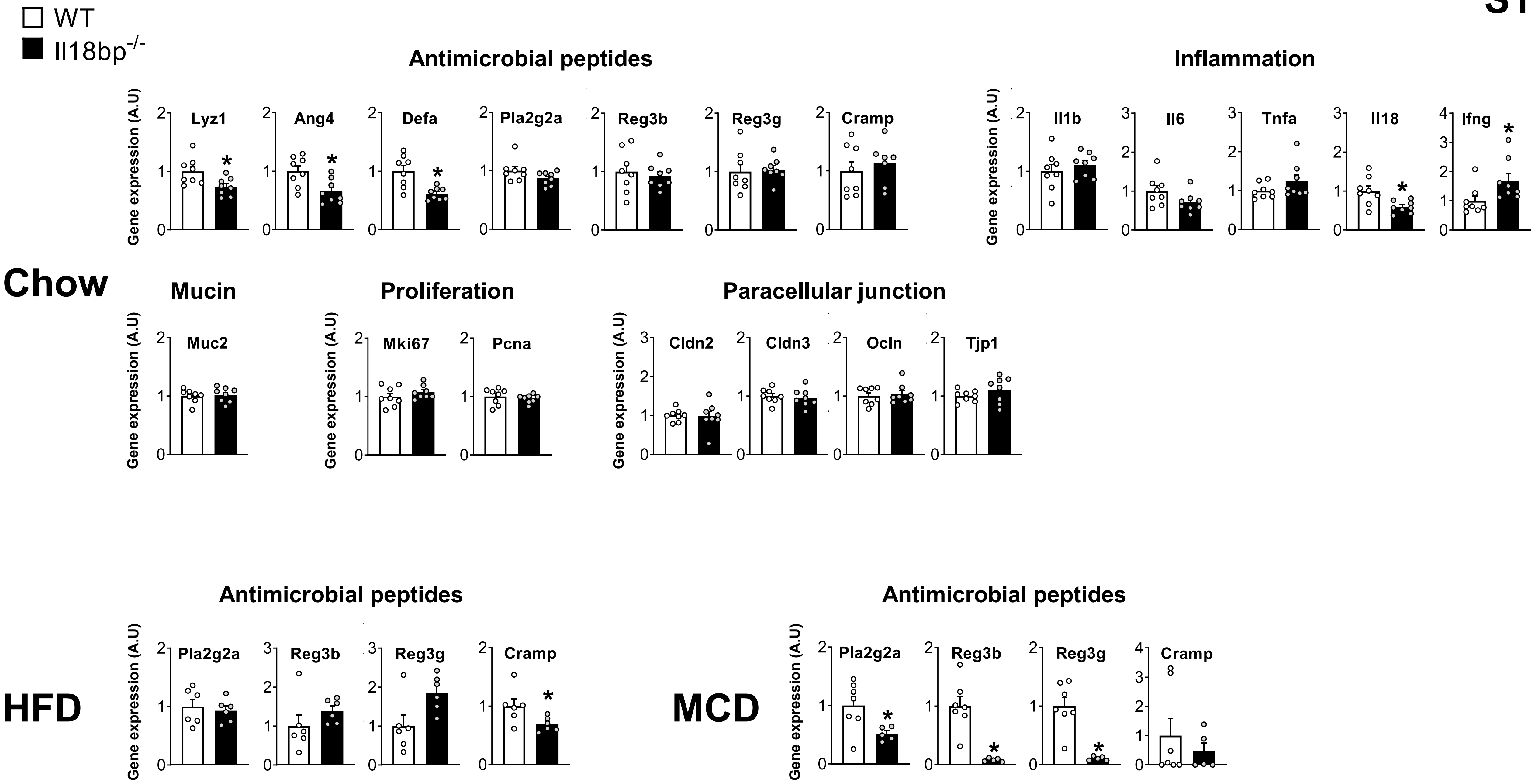

WT
Il18bp-/-
Chow
Antimicrobial peptides
Lyz1
Ang4
Defa
Pla2g2a
Reg3b
Reg3g
Cramp
Inflammation
Il1b
Il6
Tnfa
Il18
Ifng
Mucin
Muc2
Proliferation
Mki67
Pcna
Paracellular junction
Cldn2
Cldn3
Ocln
Tjp1
Gene expression (A.U)
HFD
Antimicrobial peptides
Pla2g2a
Reg3b
Reg3g
Cramp
MCD
Antimicrobial peptides
Pla2g2a
Reg3b
Reg3g
Cramp

Bacterial class

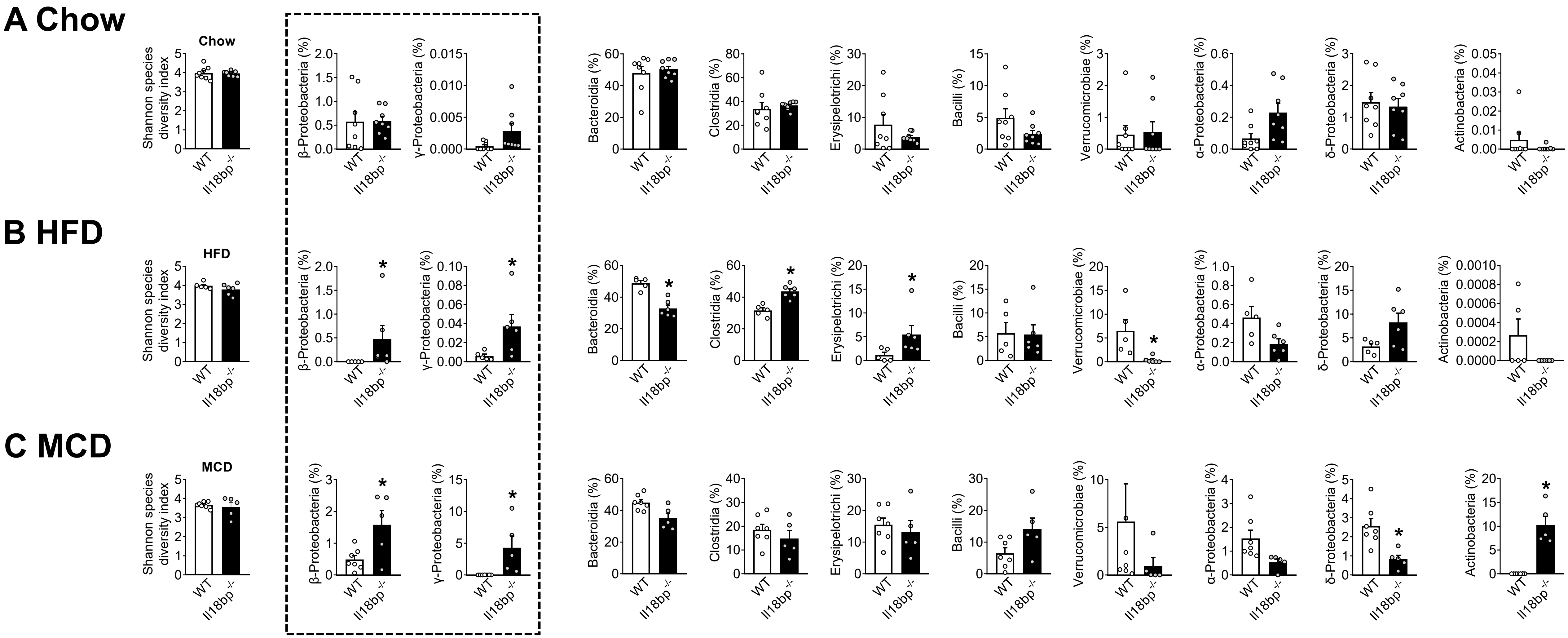

Chow diet

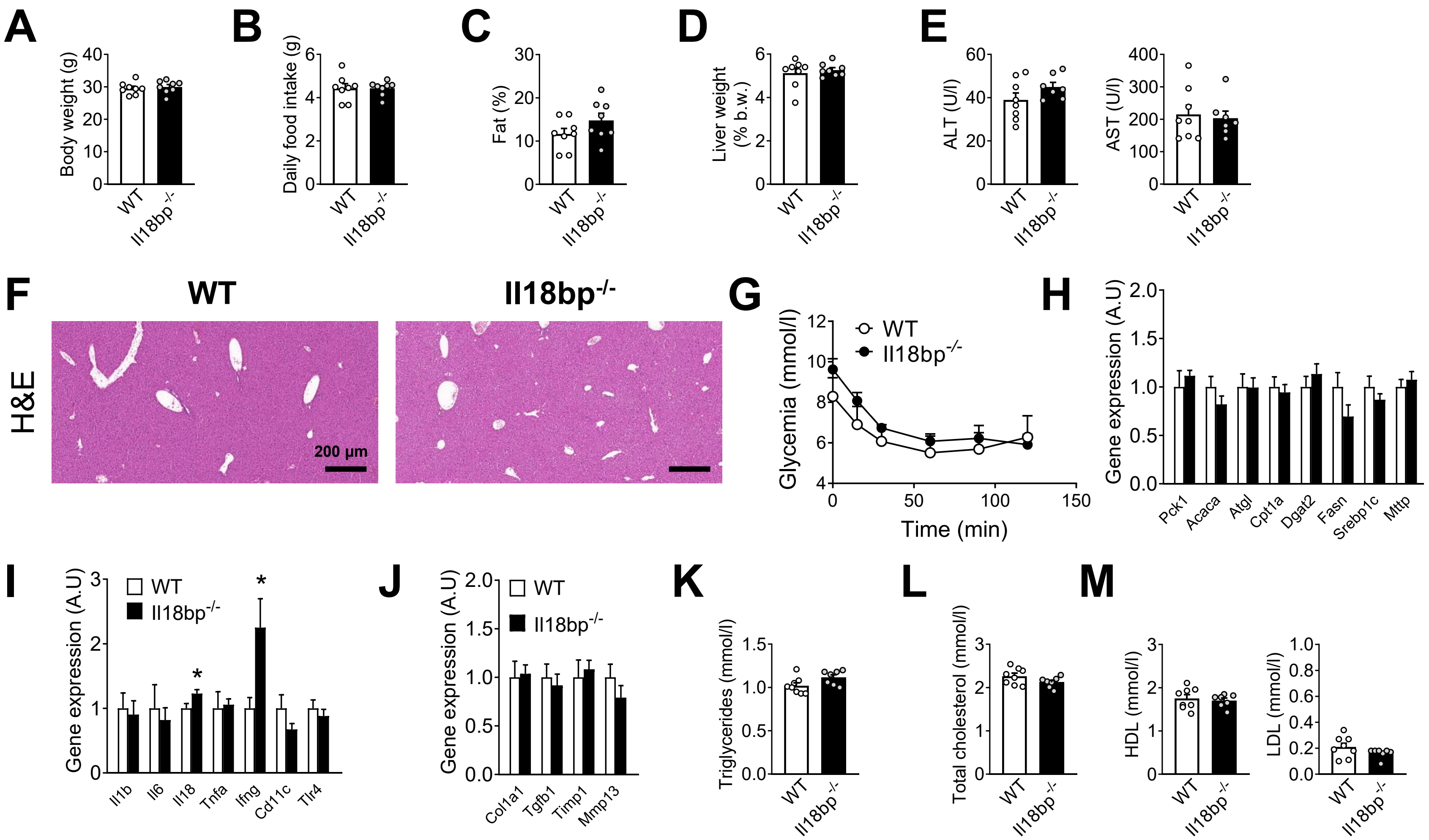
A
Body weight (g)
WT
Il18bp-/-
B
Daily food intake (g)
C
Fat (%)
D
Liver weight (% b.w.)
E
ALT (U/l)
AST (U/l)
F
WT
Il18bp-/-
H&E
200 µm
G
Glycemia (mmol/l)
Time (min)
H
Gene expression (A.U)
Pck1
Acaca
Atgl
Cpt1a
Dgat2
Fasn
Srebp1c
Mttp
I
Il1b
Il6
Il18
Tnfa
Ifng
Cd11c
Tlr4
J
Col1a1
Tgfb1
Timp1
Mmp13
K
Triglycerides (mmol/l)
L
Total cholesterol (mmol/l)
M
HDL (mmol/l)
LDL (mmol/l)

S4

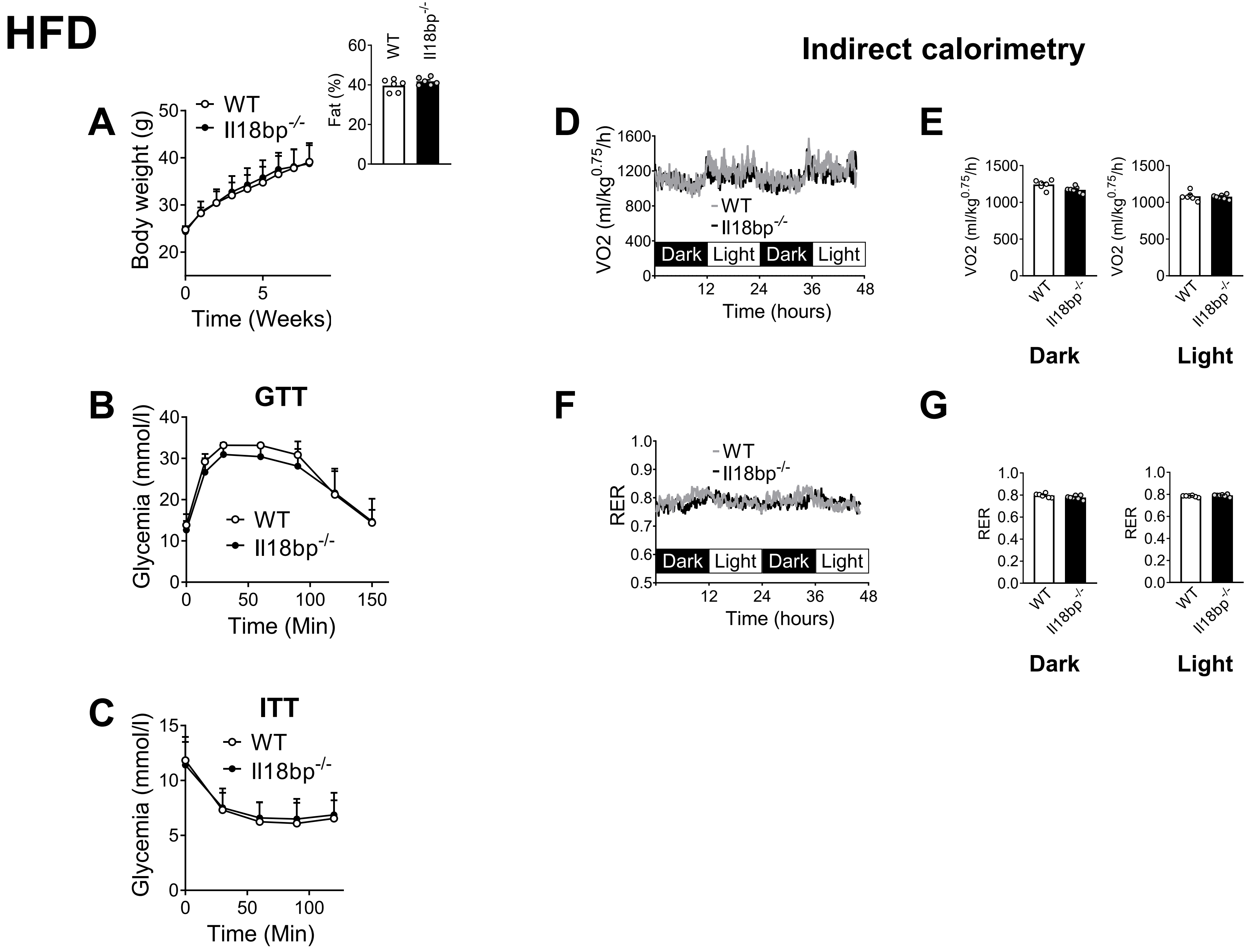
HFD
Indirect calorimetry
A
Body weight (g)
Time (Weeks)
WT
Il18bp-/-
Fat (%)
B
GTT
Glycemia (mmol/l)
Time (Min)
C
ITT
D
VO2 (ml/kg0.75/h)
Dark
Light
Time (hours)
E
F
RER
G

S5

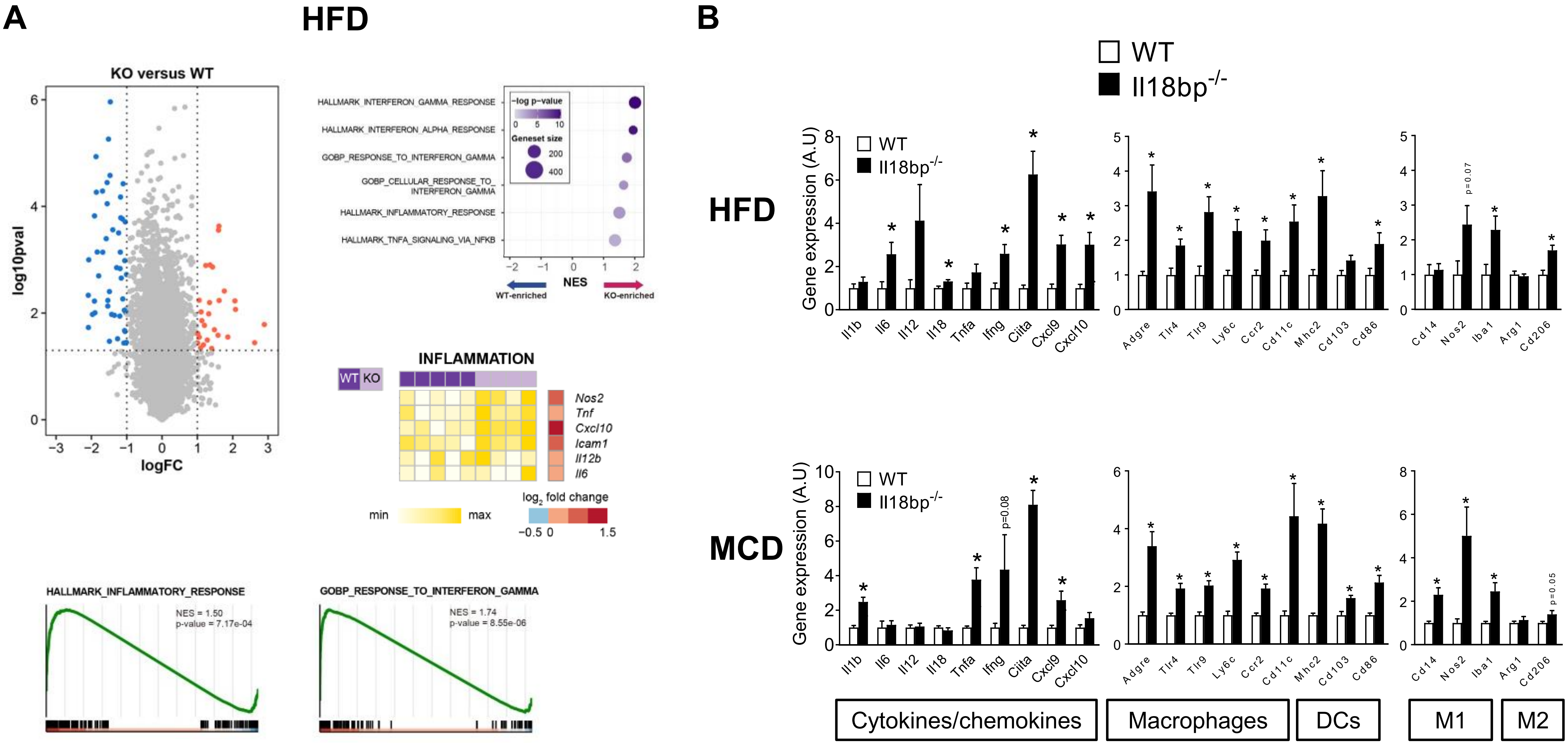
A
HFD
KO versus WT
log10pval
logFC
HALLMARK_INTERFERON_GAMMA_RESPONSE
HALLMARK_INTERFERON_ALPHA_RESPONSE
GOBP_RESPONSE_TO_INTERFERON_GAMMA
GOBP_CELLULAR_RESPONSE_TO_INTERFERON_GAMMA
HALLMARK_INFLAMMATORY_RESPONSE
HALLMARK_TNFA_SIGNALING_VIA_NFKB
-log p-value
Geneset size
NES
WT-enriched
KO-enriched
INFLAMMATION
WT KO
Nos2
Tnf
Cxcl10
Icam1
Il12b
Il6
min max
log2 fold change
HALLMARK_INFLAMMATORY_RESPONSE
NES = 1.50
p-value = 7.17e-04
GOBP_RESPONSE_TO_INTERFERON_GAMMA
NES = 1.74
p-value = 8.55e-06
B
WT
Il18bp-/-
HFD
MCD
Gene expression (A.U)
Il1b Il6 Il12 Il18 Tnfa Ifng Ciita Cxcl9 Cxcl10
Adgre Tlr4 Tlr9 Ly6c Ccr2 Cd11c Mhc2 Cd103 Cd86
Cd14 Nos2 Iba1 Arg1 Cd206
p=0.07
p=0.08
p=0.05
Cytokines/chemokines
Macrophages
DCs
M1
M2

S6

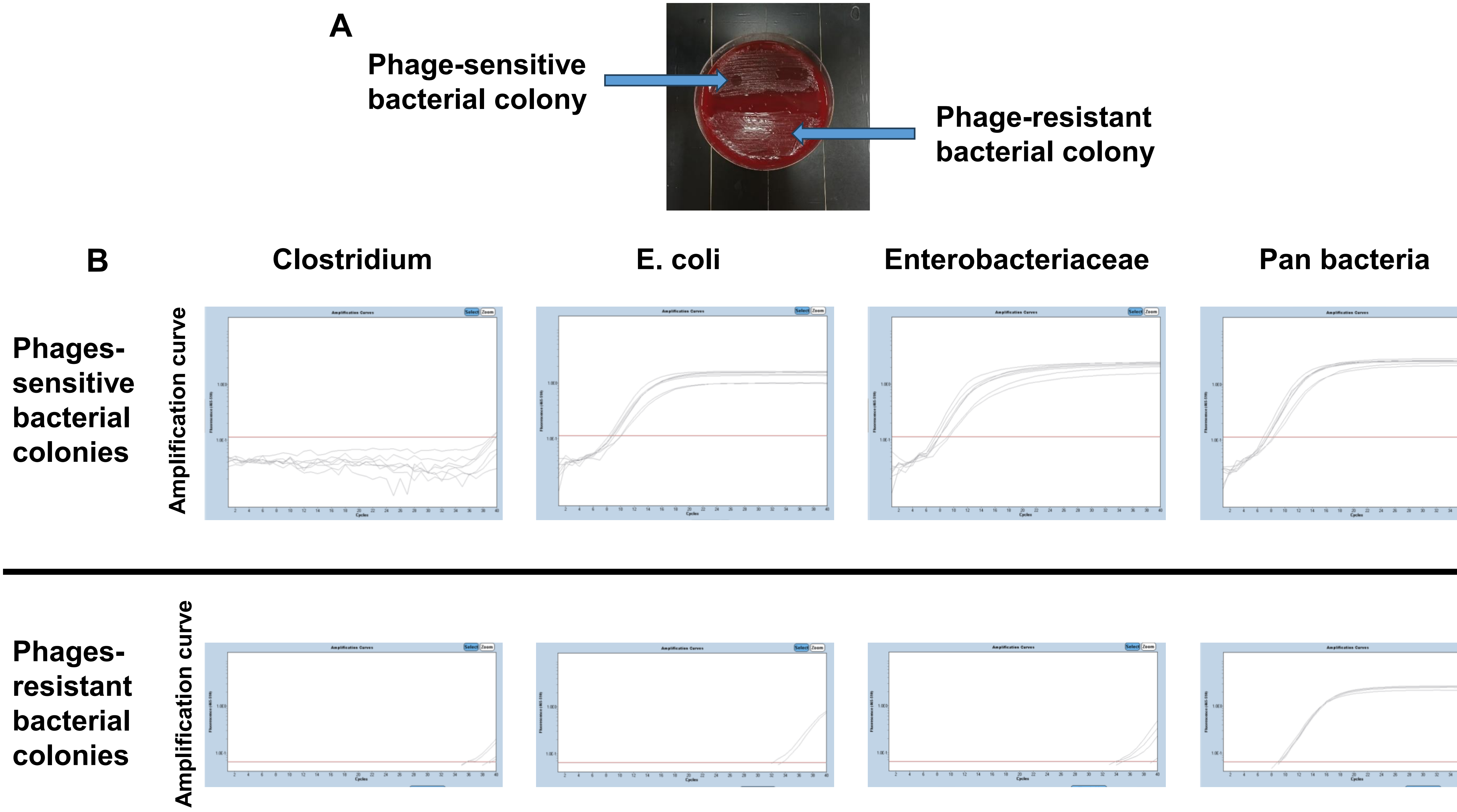
A
Phage-sensitive bacterial colony
Phage-resistant bacterial colony
B
Clostridium
E. coli
Enterobacteriaceae
Pan bacteria
Phages-sensitive bacterial colonies
Amplification curve
Phages-resistant bacterial colonies
Amplification curve

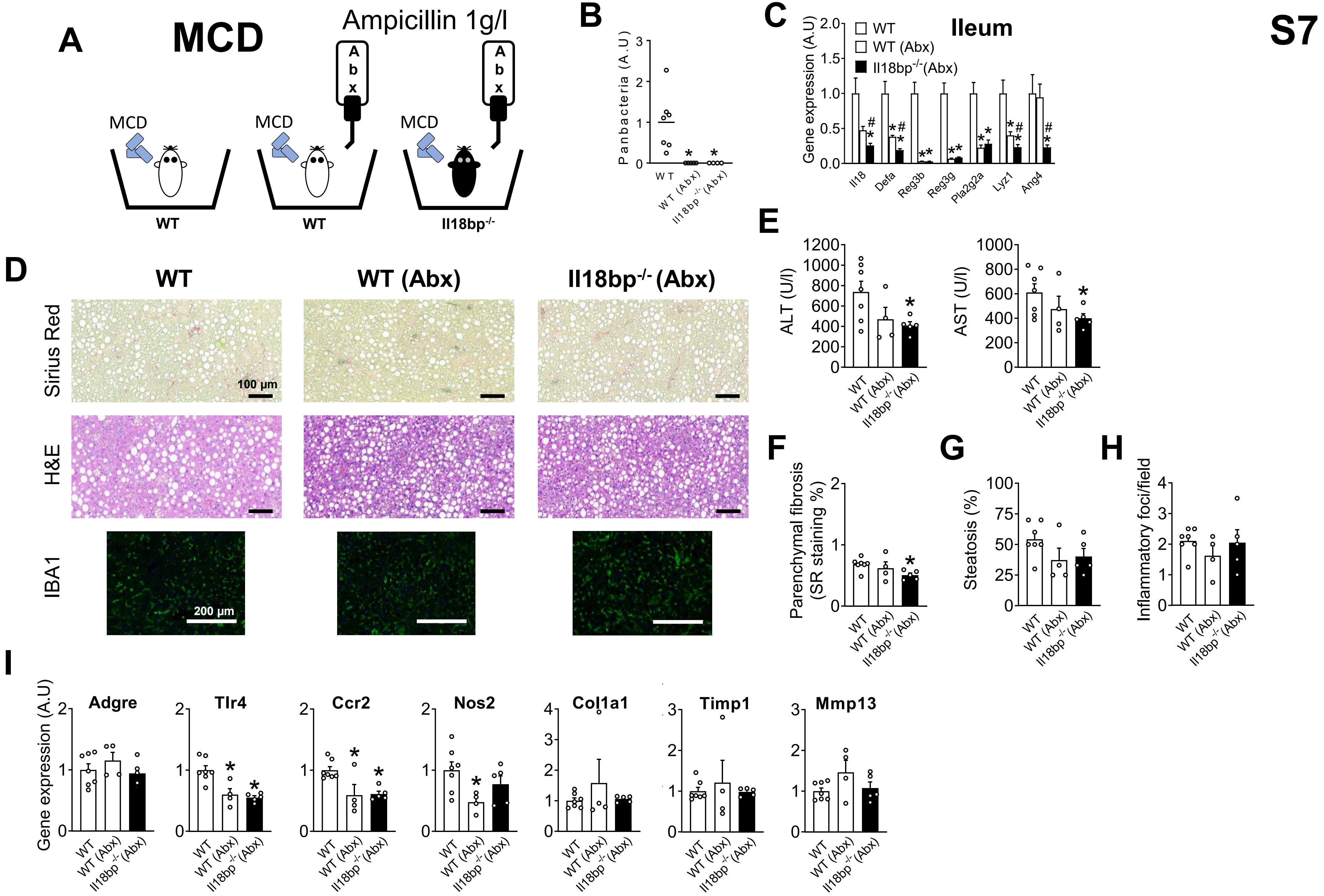

S7
A
MCD
Ampicillin 1g/l
Abx
MCD
WT
WT
Il18bp-/-
B
Panbacteria (A.U)
WT
WT (Abx)
Il18bp-/- (Abx)
C
Ileum
WT
WT (Abx)
Il18bp-/-(Abx)
Gene expression (A.U)
Il18
Defa
Reg3b
Reg3g
Pla2g2a
Lyz1
Ang4
D
WT
WT (Abx)
Il18bp-/- (Abx)
Sirius Red
H&E
IBA1
100 µm
200 µm
E
ALT (U/l)
AST (U/l)
F
Parenchymal fibrosis (SR staining %)
G
Steatosis (%)
H
Inflammatory foci/field
I
Gene expression (A.U)
Adgre
Tlr4
Ccr2
Nos2
Col1a1
Timp1
Mmp13

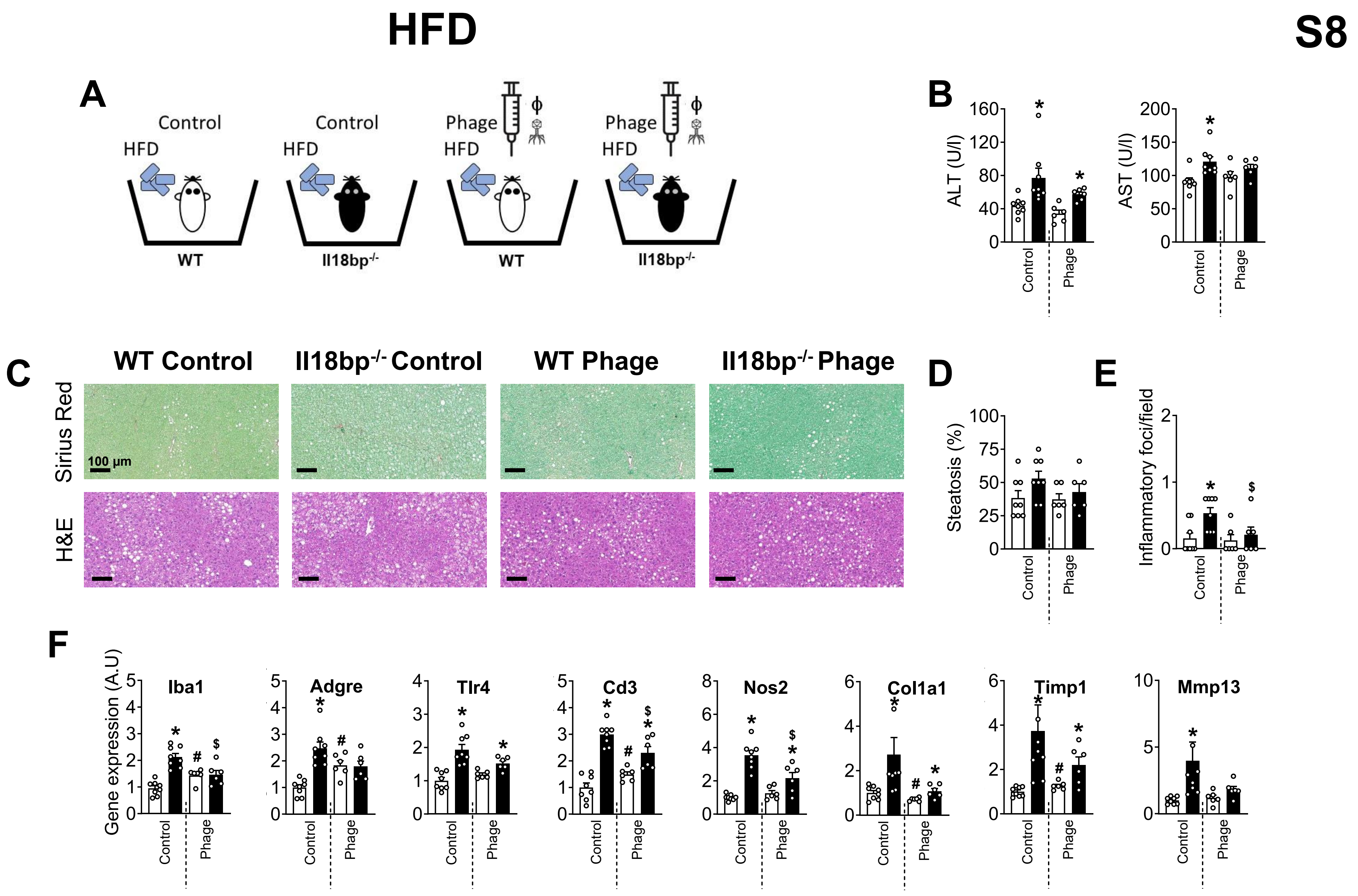
HFD
S8
A
Control
HFD
WT
Control
HFD
Il18bp-/-
Phage
HFD
WT
Phage
HFD
Il18bp-/-
B
ALT (U/l)
AST (U/l)
Control
Phage
C
WT Control
Il18bp-/- Control
WT Phage
Il18bp-/- Phage
Sirius Red
H&E
100 µm
D
Steatosis (%)
E
Inflammatory foci/field
F
Gene expression (A.U)
Iba1
Adgre
Tlr4
Cd3
Nos2
Col1a1
Timp1
Mmp13

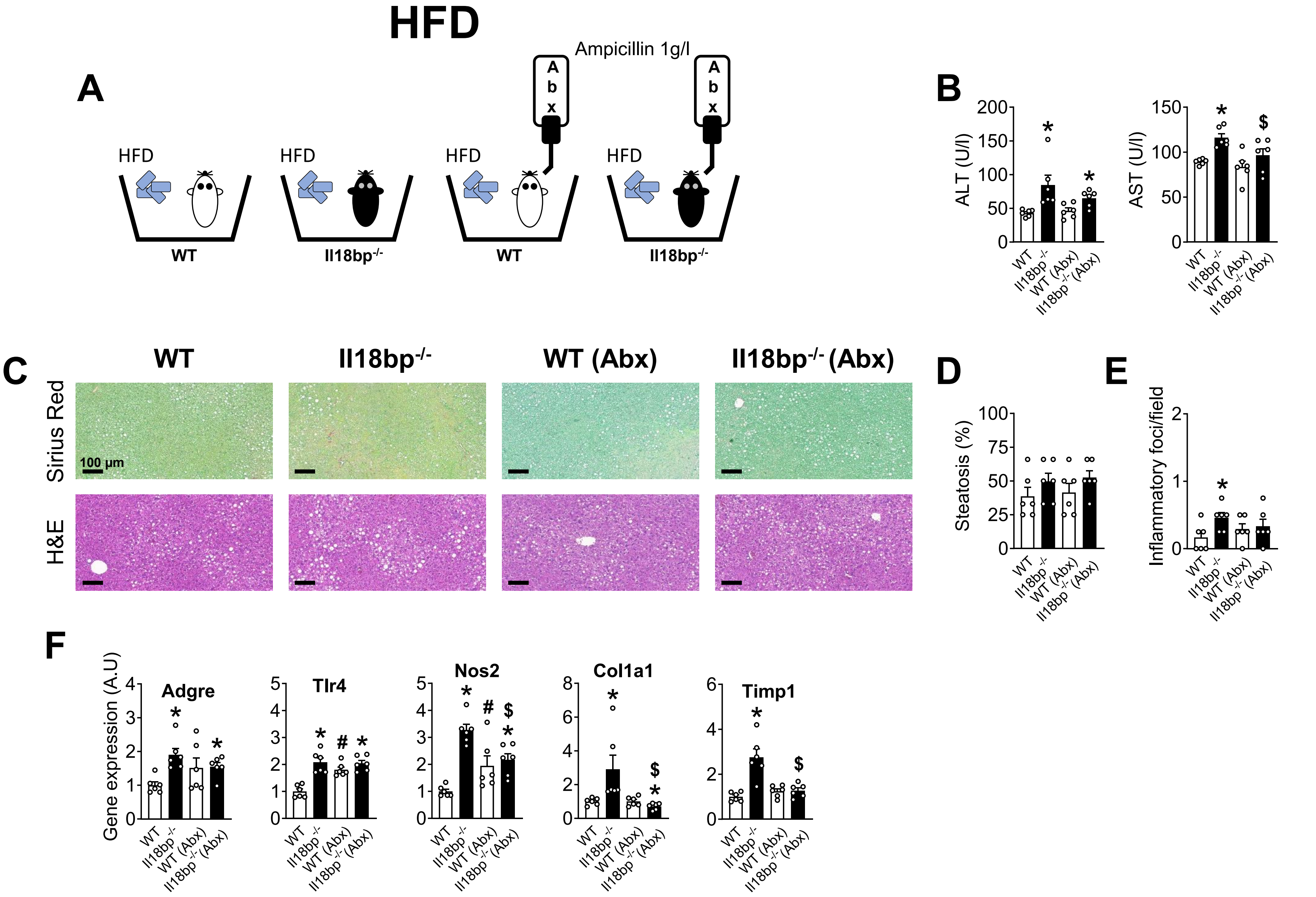

HFD
A
Ampicillin 1g/l
Abx
HFD
WT
Il18bp-/-
B
ALT (U/l)
AST (U/l)
WT
Il18bp-/-
WT (Abx)
Il18bp-/- (Abx)
C
WT
Il18bp-/-
WT (Abx)
Il18bp-/- (Abx)
Sirius Red
H&E
100 µm
D
Steatosis (%)
E
Inflammatory foci/field
F
Gene expression (A.U)
Adgre
Tlr4
Nos2
Col1a1
Timp1

S10

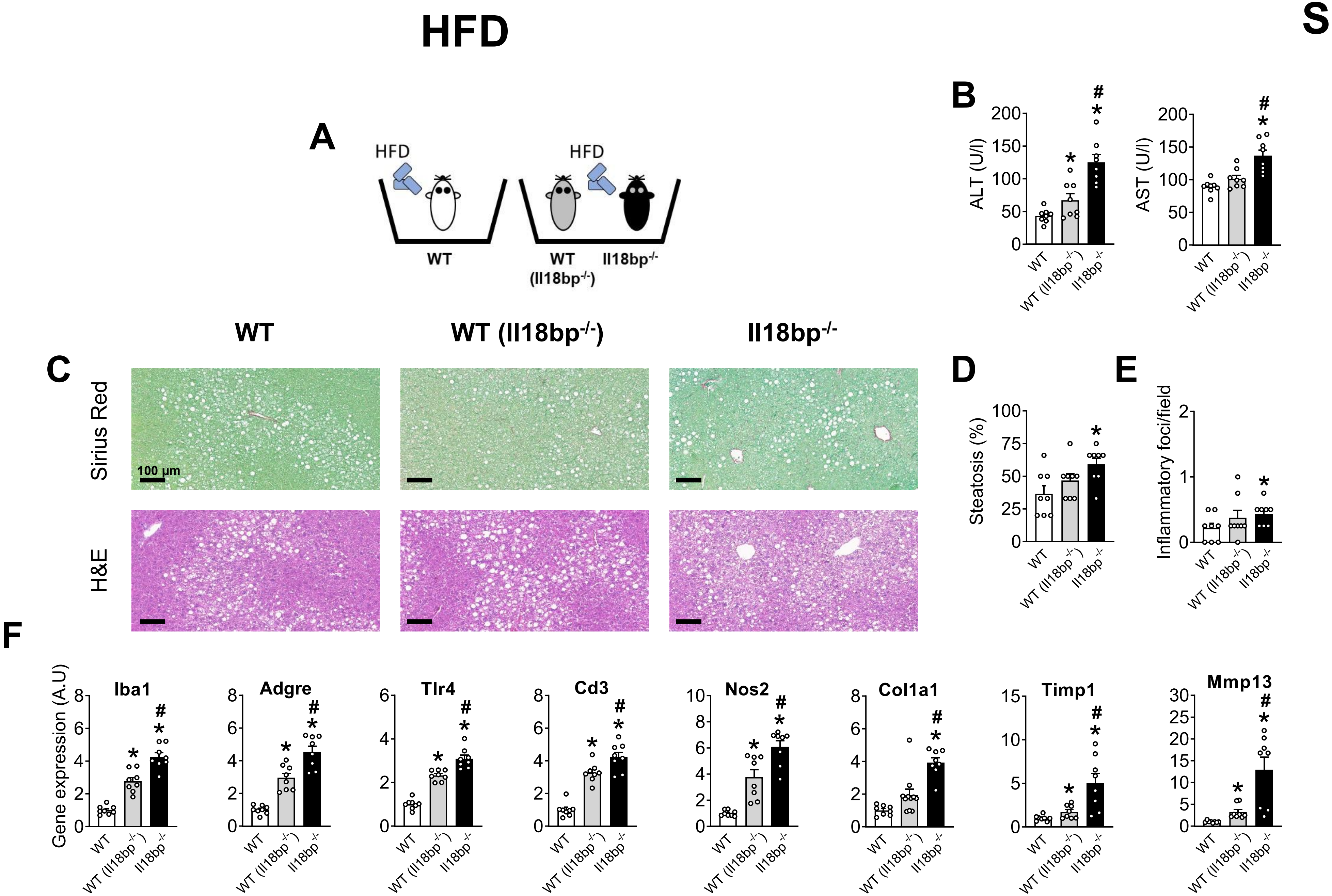
HFD
A
HFD
HFD
WT
WT (Il18bp-/-)
Il18bp-/-
B
ALT (U/l)
AST (U/l)
WT
WT (Il18bp-/-)
Il18bp-/-
C
Sirius Red
H&E
100 µm
D
Steatosis (%)
E
Inflammatory foci/field
F
Gene expression (A.U)
Iba1
Adgre
Tlr4
Cd3
Nos2
Col1a1
Timp1
Mmp13
