# Supplementary material for: Interleukin-18 binding protein deficiency results in gut microbiota dysbiosis and aggravated diet-induced MASH in mice
Source: JHEP Rep. 2025 Oct 10;8(1):101629. doi: 10.1016/j.jhepr.2025.101629 (PMC12753522; doi:10.1016/j.jhepr.2025.101629)
Supplement: Multimedia component 2 [file mmc2.docx]

**JHEP Reports**

**CTAT methods**

Tables for a “Complete, Transparent, Accurate and Timely account” (CTAT) are now mandatory for all revised submissions. The aim is to enhance the reproducibility of methods.

- Only include the parts relevant to your study
- Refer to the CTAT in the main text as ‘Supplementary CTAT Table’
- Do not add subheadings
- Add as many rows as needed to include all information
- Only include one item per row

**If the CTAT form is not relevant to your study, please outline the reasons why:**

|  |
| --- |

- 1. **Antibodies**

| **Name** | **Citation** | **Supplier** | **Cat no.** | **Clone no.** |
| --- | --- | --- | --- | --- |
| **IBA1** |  | **Wako** | **019-19741** |  |
| **IL18BP** |  | **Thermofisher** | **PA5-92981** |  |

- 1. **Cell lines**

| **Name** | **Citation** | **Supplier** | **Cat no.** | **Passage no.** | **Authentication test method** |
| --- | --- | --- | --- | --- | --- |
| **Not applicable** |  |  |  |  |  |

- 1. **Organisms**

| **Name** | **Citation** | **Supplier** | **Strain** | **Sex** | **Age** | **Overall n number** |
| --- | --- | --- | --- | --- | --- | --- |
| **WT C57BL/6J mice** |  | **Charles River** |  | **Male** | **17-24 weeks** | **104** |
| ***Il18bp^-/-^* mice** | Girard-Guyonvarc'h et Al, Blood 2018;131:1430-1441. | **Pr Cem Gabay** |  | **Male** | **17-24 weeks** | **78** |
| **Il18bp-tomato^ki/ki^ (KI) reporter mice** | Harel M et Al, J Immunol 2023;210:1790-1803. | **Pr Cem Gabay** |  | **Male** | **17-24 weeks** | **3** |

- 1. **Sequence based reagents**

| **Name** | **Sequence** | **Supplier** |
| --- | --- | --- |
| **Il18bp f** | **ACATCTGCACCTCAGACAACT** | **Microsynth** |
| **Il18bp r** | **TGGGAGGTGCTCAATGAAGGAACCA** | **Microsynth** |
| **Col1a1 f** | **AAAGACGGACTCAACGGTCTC** | **Microsynth** |
| **Col1a1 r** | **CTGAAGTCATAACCGCCACTG** | **Microsynth** |
| **Tgfb1 f** | **AAGTTGGCATGGTAGCCCTT** | **Microsynth** |
| **Tgfb1 r** | **GCCCTGGATACCAACTATTGC** | **Microsynth** |
| **Timp1 f** | **CCTTGCAAACTGGAGAGTGAC** | **Microsynth** |
| **Timp1 r** | **AAGCAAAGTGACGGCTCTGGT** | **Microsynth** |
| **Mmp13 f** | **AGGCCTTCAGAAAAGCCTTC** | **Microsynth** |
| **Mmp13 r** | **TCCTTGGAGTGATCCAGACC** | **Microsynth** |
| **Il1b f** | **GATGAAGGGCTGCTTCCAAAC** | **Microsynth** |
| **Il1b r** | **GTGCTGCTGCGAGATTTGAA** | **Microsynth** |
| **Il6 f** | **CCTTCAGAGAGATACAGAAACTCTAATTCA** | **Microsynth** |
| **Il6 r** | **CTGTTAGGAGAGCATTGGAAATTG** | **Microsynth** |
| **Il12 f** | **GGAAGCACGGCAGCAGAATA** | **Microsynth** |
| **Il12 r** | **AACTTGAGGGAGAAGTAGGAATGG** | **Microsynth** |
| **Il18 f** | **CAGGCCTGACATCTTCTG** | **Microsynth** |
| **Il18 r** | **CTGACATGGCAGCCATT** | **Microsynth** |
| **Tnfa f** | **CCACGCTCTTCTGTCTACTGAACTT** | **Microsynth** |
| **Tnfa r** | **GATGAGAGGGAGGCCATTTG** | **Microsynth** |
| **Ifng f** | **CATCAGCAACAACATAAGCGTCA** | **Microsynth** |
| **Ifng r** | **CTCCTTTTCCGCTTCCTGA** | **Microsynth** |
| **Ciita f** | **CAGCACTCAGAAGCACGGG** | **Microsynth** |
| **Ciita r** | **ATCCATGGTGGCACACAGACT** | **Microsynth** |
| **Cxcl9 f** | **CGAGGCACGATCCACTACAA** | **Microsynth** |
| **Cxcl9 r** | **AGGCAGGTTTGATCTCCGTT** | **Microsynth** |
| **Cxcl10 (Ip-10) f** | **CCAAGTGCTGCCGTCATTTTC** | **Microsynth** |
| **Cxcl10 (Ip-10) r** | **GGCTCGCAGGGATGATTTCAA** | **Microsynth** |
| **Cd14 f** | **CTCTGTCCTTAAAGCGGCTTAC** | **Microsynth** |
| **Cd14 r** | **GTTGCGGAGGTTCAAGATGTT** | **Microsynth** |
| **Nos2 f** | **ATTCACAGCTCATCCGGTACG** | **Microsynth** |
| **Nos2 r** | **GGATCTTGACCATCAGCTTGC** | **Microsynth** |
| **Iba1 (Aif1) f** | **CAGACTGCCAGCCTAAGACA** | **Microsynth** |
| **Iba1 (Aif1) r** | **AGGAATTGCTTGTTGATCCC** | **Microsynth** |
| **Arg1 f** | **CTCCAAGCCAAAGTCCTTAGAG** | **Microsynth** |
| **Arg1 r** | **AGGAGCTGTCATTAGGGACATC** | **Microsynth** |
| **Cd206 f** | **CAGGTGTGGGCTCAGGTAGT** | **Microsynth** |
| **Cd206r** | **TGTGGTGAGCTGAAAGGTGA** | **Microsynth** |
| **Il4 f** | **AGATGGATGTGCCAAACGTCCTCA** | **Microsynth** |
| **Il4 r** | **AATATGCGAAGCACCTTGGAAGCC** | **Microsynth** |
| **Il5 f** | **TCCTTGCAGTGTGAATGAGAG** | **Microsynth** |
| **Il5 r** | **CCCTGATACCTGAATAACATCCC** | **Microsynth** |
| **Il10 f** | **TGTGAAAATAAGAGCAAGGCAGTG** | **Microsynth** |
| **Il10 r** | **CATTCATGGCCTTGTAGACACC** | **Microsynth** |
| **Il13 f** | **CCTCTGACCCTTAAGGAGCTTAT** | **Microsynth** |
| **Il13 r** | **CGTTGCACAGGGGAGTCT** | **Microsynth** |
| **Tlr4 f** | **GCTCCTGGCTAGGACTCTGAT** | **Microsynth** |
| **Tlr4 r** | **CTGATCCATGCATTGGTAGGT** | **Microsynth** |
| **Atgl (**Pnpla2**) f** | **GTCCTTCACCATCCGCTTGTT** | **Microsynth** |
| **Atgl (**Pnpla2**) r** | **CTCTTGGCCCTCATCACCAG** | **Microsynth** |
| **Cpt1a f** | **AACCCAGTGCCTTAACGATG** | **Microsynth** |
| **Cpt1a r** | **GAACTGGTGGCCAATGAGAT** | **Microsynth** |
| **Dgat2 f** | **TGGGCCTTGGTGGTTTCTTAC** | **Microsynth** |
| **Dgat2 r** | **GACTGCCCTTGCCCAGCTA** | **Microsynth** |
| **Fasn f** | **CATGACCTCGTGATGAACGTGT** | **Microsynth** |
| **Fasn r** | **CGGGTGAGGACGTTTACAAAG** | **Microsynth** |
| **Ly6c f** | **GCAGTGCTACGAGTGCTATGG** | **Microsynth** |
| **Ly6c r** | **ACTGACGGGTCTTTAGTTTCCTT** | **Microsynth** |
| **Cd3 f** | **ATGCGGTGGAACACTTTCTGG** | **Microsynth** |
| **Cd3 r** | **GCACGTCAACTCTACACTGGT** | **Microsynth** |
| **Defa f** | **CTATCTCCTTTGGAGGCCAAG** | **Microsynth** |
| **Defa r** | **ATTTCTGCAGGTCCCAAAAAC** | **Microsynth** |
| **Lyz1 f** | **GCCAAGGTCTACAATCGTTGTGAGTTG** | **Microsynth** |
| **Lyz1 r** | **CAGTCAGCCAGCTTGACACCACG** | **Microsynth** |
| **Ang4 f** | **CTCTGGCTCAGAATGAAAGGTACGA** | **Microsynth** |
| **Ang4 r** | **GAAATCTTTAAAGGCTCGGTACCC** | **Microsynth** |
| **Pla2g2a f** | **GACCGGTGCTGTGTTACTCAT** | **Microsynth** |
| **Pla2g2a r** | **GCCGTTTCTGACAGGAGTTCT** | **Microsynth** |
| **Reg3b f** | **ATGCTGCTCTCCTGCCTGATG** | **Microsynth** |
| **Reg3b r** | **CTAATGCGTGCGGAGGGTATATTC** | **Microsynth** |
| **Reg3g f** | **TTCCTGTCCTCCATGATCAAA** | **Microsynth** |
| **Reg3g r** | **CATCCACCTCTGTTGGGTTC** | **Microsynth** |
| **Muc2 f** | **ATGCCCACCTCCTCAAAGAC** | **Microsynth** |
| **Muc2 r** | **GTAGTTTCCGTTGGAACAGTGAA** | **Microsynth** |
| **Cldn2 f** | **GTCATCGCCCATCAGAAGAT** | **Microsynth** |
| **Cldn2 r** | **ACTGTTGGACAGGGAACCAG** | **Microsynth** |
| **Cldn3 f** | **TCATCGGCAGCAGCATCATCAC** | **Microsynth** |
| **Cldn3 r** | **ACGATGGTGATCTTGGCCTTGG** | **Microsynth** |
| **Ocln f** | **TACTCCTCCAATGGCAAAGTG** | **Microsynth** |
| **Ocln r** | **CCCACCTGTCGTGTAGTCTGT** | **Microsynth** |
| **Tjp1 f** | **GCAATGGTTAACGGAGTTTCA** | **Microsynth** |
| **Tjp1 r** | **TGGGTGACTTACAGGGATCTG** | **Microsynth** |
| **Adgre f** | **CTGGGATCCTACAGCTGCTC** | **Microsynth** |
| **Adgre r** | **AGGAGCCTGGTACATTGGTG** | **Microsynth** |
| **β-proteobact f** | **AACGCGAAAAACCTTACCTACC** | **Microsynth** |
| **β-proteobact r** | **TGCCCTTTCGTAGCAACTAGTG** | **Microsynth** |
| **γ-proteobact f** | **TCGTCAGCTCGTGTYGTGA** | **Microsynth** |
| **γ-proteobact r** | **CGTAAGGGCCATGATG** | **Microsynth** |
| **Enterobact f** | **TGCCGTAACTTCGGGAGAAGGCA** | **Microsynth** |
| **Enterobact r** | **TCAAGGCTCAATGTTCAGTGTC** | **Microsynth** |
| **E.coli f** | **CATGCCGCGTGTATGAAGAA** | **Microsynth** |
| **E.coli r** | **CGGGTAACGTCAATGAGCAAA** | **Microsynth** |
| **Pck1 f** | **CACCATCACCTCCTGGAAGA** | **Microsynth** |
| **Pck1 r** | **GGGTGCAGAATCTCGAGTTG** | **Microsynth** |
| **Acaca f** | **AGGAAGATGGCGTCCGCTCTG** | **Microsynth** |
| **Acaca r** | **GGTGAGATGTGCTGGGTCAT** | **Microsynth** |
| **Srebp1c f** | **GGAGCCATGGATTGCACATT** | **Microsynth** |
| **Srebp1c r** | **GGCCCGGGAAGTCACTGT** | **Microsynth** |
| **Mttp f** | **CTCTTGGCAGTGCTTTTTCTCT** | **Microsynth** |
| **Mttp r** | **GAGCTTGTATAGCCGCTCATT** | **Microsynth** |
| **Cd11c f** | **CTGGATAGCCTTTCTTCTGCTG** | **Microsynth** |
| **Cd11c r** | **GCACACTGTGTCCGAACTCA** | **Microsynth** |
| **Tlr9 f** | **GAATCCTCCATCTCCCAACAT** | **Microsynth** |
| **Tlr9 r** | **TTCAGCTCACAGGGTAGGAAG** | **Microsynth** |
| **Ccr2 f** | **ATCCACGGCATACTATCAACATC** | **Microsynth** |
| **Ccr2 r** | **CAAGGCTCACCATCATCGTAG** | **Microsynth** |
| **Mhc2 f** | **AGCCCCATCACTGTGGAGT** | **Microsynth** |
| **Mhc2 r** | **GATGCCGCTCAACATCTTGC** | **Microsynth** |
| **Cd103 f** | **CCTGTGCAGCATGTAAAAGAATG** | **Microsynth** |
| **Cd103 r** | **CAAGGATCGGCAGTTCAGATAC** | **Microsynth** |
| **Cd86 f** | **TCTCCACGGAAACAGCATCT** | **Microsynth** |
| **Cd86 r** | **CTTACGGAAGCACCCATGAT** | **Microsynth** |
| **Camp f** | **TCTCTACCGTCTCCTGGACCT** | **Microsynth** |
| **Camp r** | **TCACTCGGAACCTCACAGACT** | **Microsynth** |
| **MKi67 f** | **CCTTTGCTGTCCCCGAAGA** | **Microsynth** |
| **MKi67 r** | **GGCTTCTCATCTGTTGCTTCCT** | **Microsynth** |
| **Pcna f** | **CAAGTGGAGAGCTTGGCAATGG** | **Microsynth** |
| **Pcna r** | **GCAAACGTTAGGTGAACAGGCTC** | **Microsynth** |
| **Rps29 f** | **GCCAGGGTTCTCGCTCTTG** | **Microsynth** |
| **Rps29 r** | **GGCACATGTTCAGCCCGTAT** | **Microsynth** |
| **Pan-bact f** | **GCAGGCCTAACACATGCAAGTC** | **Microsynth** |
| **Pan-bact r** | **CTGCTGCCTCCCGTAGGAGT** | **Microsynth** |

- 1. **Biological samples**

| **Description** | **Source** | **Identifier** |
| --- | --- | --- |
| **Not applicable** |  |  |

- 1. **Deposited data**

| **Name of repository** | **Identifier** | **Link** |
| --- | --- | --- |
| European Nucleotide Archive (ENA) | study number: PRJEB60286 | https://www.ebi.ac.uk/ena/browser/view/PRJEB60286 |

- 1. **Software**

| **Software name** | **Manufacturer** | **Version** |
| --- | --- | --- |
| ZEN 3.1 blue edition | Zeiss |  |
| Axio Scan.Z1 slide scanner software | Zeiss |  |
| QuPath software | Open access | V0.4.3 <https://qupath.github.io> |
| Image J | NIH | Fiji 1.54f |
| GraphPad Prism software |  | 9.5.1 |
| Endnote X9 | Clarivate |  |
|  |  |  |

- 1. **Other (e.g. drugs, proteins, vectors etc.)**

| ampicillin | Ratiopharm |  |
| --- | --- | --- |
| E.coli-Proteus bacteriophage solution | Microgen (Russia) |  |
| High-Fat diet (HFD) (D12492) | Research Diets |  |
| Methionine choline deficient (MCD)-diet (A02082002BR) | Research Diets |  |
| Chow diet SAFE 150 | SAFE DIETS |  |

- 1. **Please provide the details of the corresponding methods author for the manuscript:**

| **Emmanuel Somm**  Service of Endocrinology/Geneva University Hospitals  Department of Cell Physiology and Metabolism, University of Geneva, Switzerland.  Centre Médical Universitaire, 1 rue Michel Servet, 1206 Genève, SUISSE  Emmanuel.somm@unige.ch |
| --- |

**2.0 Please confirm for randomised controlled trials all versions of the clinical protocol are included in the submission. These will be published online as supplementary information.**

| **Not applicable** |
| --- |
